# New Microporous Lanthanide Organic Frameworks. Synthesis, Structure, Luminescence, Sorption, and Catalytic Acylation of 2-Naphthol

**DOI:** 10.3390/molecules25133055

**Published:** 2020-07-03

**Authors:** Dana Bejan, Lucian Gabriel Bahrin, Sergiu Shova, Narcisa Laura Marangoci, Ülkü Kökҫam-Demir, Vasile Lozan, Christoph Janiak

**Affiliations:** 1 “Petru Poni” Institute of Macromolecular Chemistry, Romanian Academy, 700487 Iasi, Romania; bahrin.lucian@icmpp.ro (L.G.B.); shova@icmpp.ro (S.S.); nmarangoci@icmpp.ro (N.L.M.); 2 Institut für Anorganische Chemie und Strukturchemie, Heinrich-Heine Universität Düsseldorf, Universitätsstr. 1, D 40225 Düsseldorf, Germany; Uelkue.Koekcam@uni-duesseldorf.de; 3 Institute of Chemistry of MECR, Academiei str. 3, MD2028 Chisinau, Moldova

**Keywords:** metal-organic frameworks, lanthanides, luminescence, sorption, catalysis

## Abstract

New metal-organic frameworks (MOF) with lanthanum(III), cerium(III), neodymium(III), europium(III), gadolinium(III), dysprosium(III), and holmium(III)] and the ligand precursor *1,3,5-*tris(*4*-carboxyphenyl)-*2,4,6*-trimethylbenzene (H_3_L) were synthesized under solvothermal conditions. Single crystal x-ray analysis confirmed the formation of three-dimensional frameworks of [LnL(H_2_O)_2_]_n_·xDMF·yH_2_O for Ln = La, Ce, and Nd. From the nitrogen sorption experiments, the compounds showed permanent porosity with Brunauer-Emmett-Teller (BET) surface areas of about 400 m^2^/g, and thermal stability up to 500 °C. Further investigations showed that these Ln-MOFs exhibit catalytic activity, paving the way for potential applications within the field of catalysis.

## 1. Introduction

**Figure 1:**
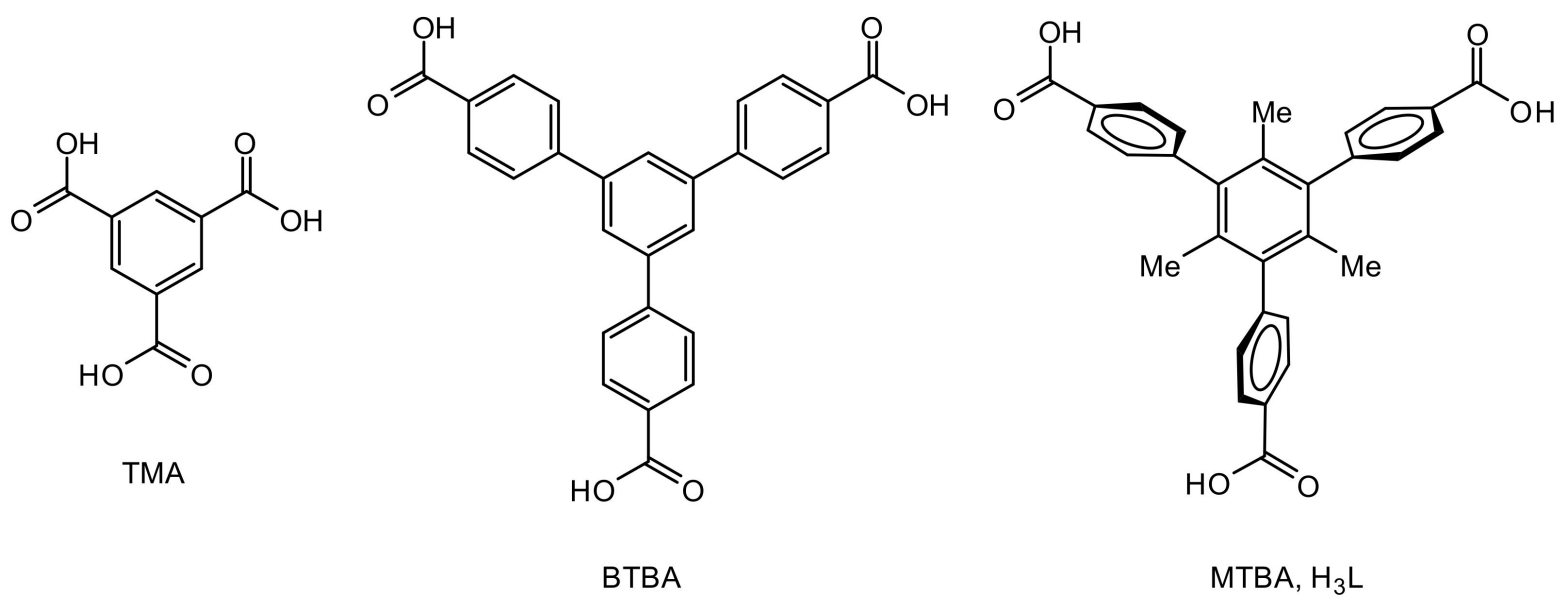
Tritopic linkers used in the construction of metal-organic frameworks (MOFs).

Metal-organic frameworks (MOFs) form a class of porous materials that have been intensively studied in the last decade [1,2,3,4,5,6]. MOFs provide diverse and easily modifiable architectures and topologies and play important roles in synthetic chemistry [7], catalysis [2,8,9,10], molecular separation [11], magnetism [12,13], optics [14,15] possess luminescence [16,17], and are used as potential drug delivery carriers [18,19,20]. Mostly, MOFs are of interest [21,22,23] as microporous materials in gas/solvent sorption applications due to their high porosity and tunable pore structure. Amongst the intensely studied carboxylate ligands is 1,3,5-benzenetricarboxylate, the trianion of trimesic acid (TMA) [24,25]. Extended derivatives of TMA, namely 1,3,5-benzenetribenzoic acid (BTBA) and mesitylenetribenzoic acid (MTBA) are also exploited as organic spacers in MOFs (see Figure 1).

MOFs designed with these ligands exhibit properties and functions that are attractive to researchers due to their rigidity and trifunctional identity [26,27,28]. Even though the BTBA and MTBA systems are at first glance quite similar, their geometries differ markedly. The three methyl groups in MTBA induce steric hindrance and lead to a structure in which the three carboxylate groups are almost 90° out of plane with the central benzene moiety. Attempts to crystallize BTBA and establish its crystal structure have been unsuccessful thus far [29], while hydrogen bond-mediated self-assembly of MTBA was found to result in honeycomb networks with enlarged pores of ca. 24–26 Å in diameter, which can accommodate K^+^-coordinated 18-crown-6 and dibenzo-18-crown-6, as shown by a series of x-ray diffraction structures reported recently [30]. BTBA and MTBA were used in the development of new design strategies for MOF materials with the desired porosity, pore size, and functionality [31]. Mixed-ligand Archimedian polyhedron-based MOFs assembled from equilateral triangles (BTBA or MTBA) and squares (porphyrin) were shown to be efficient photocatalysts for CO_2_ reduction upon visible light irradiation [31]. Mesoporous MOFs consisting of M_6_ nodes (M = Zr, Hf, Ce, Th) and MTBA as a tritopic linker have been prepared and used as supporting materials for a vanadium catalyst in the aerobic oxidation of 4-methylbenzyl alcohol [32].

Coordination geometry flexibility and high coordination numbers make lanthanide-based MOFs a promising class of porous materials with useful properties such as luminescence, thermal stability, magnetism, and catalytic behavior [26,33,34,35,36]. MTBA possesses intrinsic fluorescence. Even though a number of complexes with MTBA as a linker have been documented in the literature [28,30,31,37,38,39,40,41], lanthanide(III)-based MOFs with this tritopic ligand have not been reported as yet. 

Herein, we report on the synthesis and characterization of a series of lanthanide-based MOFs of general formula [LnL(H_2_O)_2_]_n_ where Ln = La (**1**), Ce (**2**), Nd (**3**), Eu (**4**), Gd (**5**), Dy (**6**), and Ho (**7**), and *1,3,5-*tris(*4*-carboxyphenyl)-*2,4,6*-trimethylbenzene (H_3_L) is the linker. The aim of this work was the synthesis of new MOFs with the tribasic tricarboxylate H_3_L ligand (MTBA) and the investigation of their physicochemical properties, crystal structures, thermal, and luminescence properties. Additionally, for these new Ln-MOFs, their potential catalytic activity was established for O-acylation reactions.

## 2. Results and Discussion

The linker precursor *1,3,5-*tris(*4*-carboxyphenyl)-*2,4,6*-trimethylbenzene (H_3_L), also called mesitylenetribenzoic acid (MTBA), previously reported by Bajpai et al. [30], was prepared in moderate yield (50%) as described in the Supplementary Materials. The structure and purity of H_3_L were confirmed by IR and NMR spectroscopy. The lanthanide complexes **1**–**7** were prepared by the solvothermal reaction of H_3_L with Ln(NO_3_)_3_·6H_2_O [Ln = La; Ce; Nd; Eu; Gd; Dy; Ho]. The optical microscopy images (Figure S1) of all lanthanide complexes are given in the SM. To optimize the reaction conditions, different solvents and mixtures were used (DMF, DMSO, H_2_O, and ethanol). In addition, different reaction temperatures and times were used in order to obtain crystalline products suitable for x-ray diffraction. The optimal conditions were found to be an EtOH/H_2_O/DMF solvent mixture, a reaction temperature of 80 °C, and a reaction time of one to five days (see Table S1, in the SM). 

The chemical robustness of the Ln-MOF **3** was exemplarily checked by immersing in a 2 mol/L solution of NaOH and HNO_3_, respectively, for one day. Compound **3** (10 mg) was suspended in 2 mL of acidic/basic solution for 24 h, filtered off, washed, dried in air, and investigated by PXRD analysis. As can be seen in Figure S14 (SM), compound **3** decomposed in 2 mol/L of aqueous acid or base. The Ln-MOFs **1**–**7** were, however, stable in air and solvent (ethanol, DMF, H_2_O-mother liquor) for more than one week. Powder x-ray diffraction patterns recorded after two weeks showed that these compounds are unaffected by the solvents.

### 2.1. Structural Characterization 

**Figure 2:**
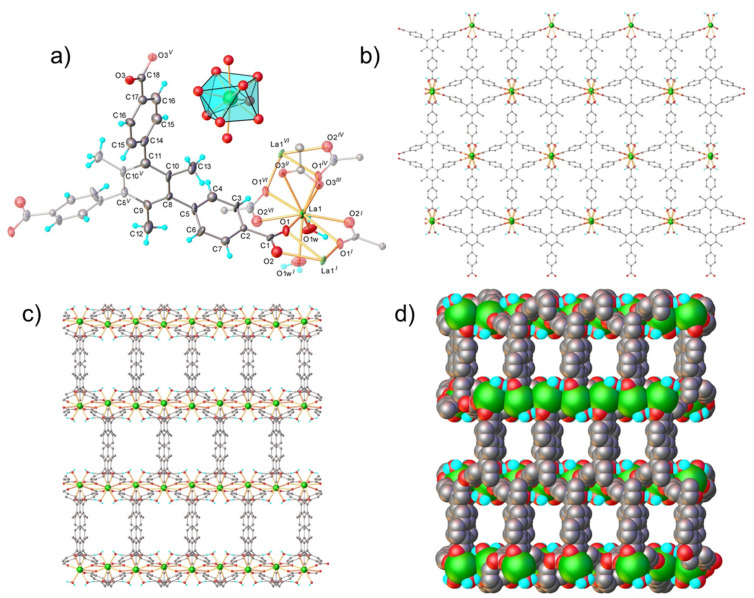
Crystal structure of compound [LaL(H_2_O)_2_]_n_ (**1**). Extended view of the asymmetric part and coordination environment around La in **1** along with the atom labelling scheme and thermal ellipsoids at the 40% probability level. Symmetry generated fragments are drawn with 50% transparency (**a**). Crystal structure packing viewed along the *a* axis (**b**). View of the crystal structure showing formation of the channels along the *b* axis (**c**). Space filling representation for (**d**). Symmetry codes: I = 1 − *x*, 1 − *y*, 1 − *z*; II = 1.5 − *x*, 0.5 + *y*, 0.5 − *z*; III = *x*, 0.5 − *y*, 0.5 − *z*; IV = 2 − *x*, 1 − *y*, 1 − *z*; V = 1.5 − *x*, − *y*, *z*; 0.5 + *x*, *y*, 1 − *z*.

**Figure 3:**
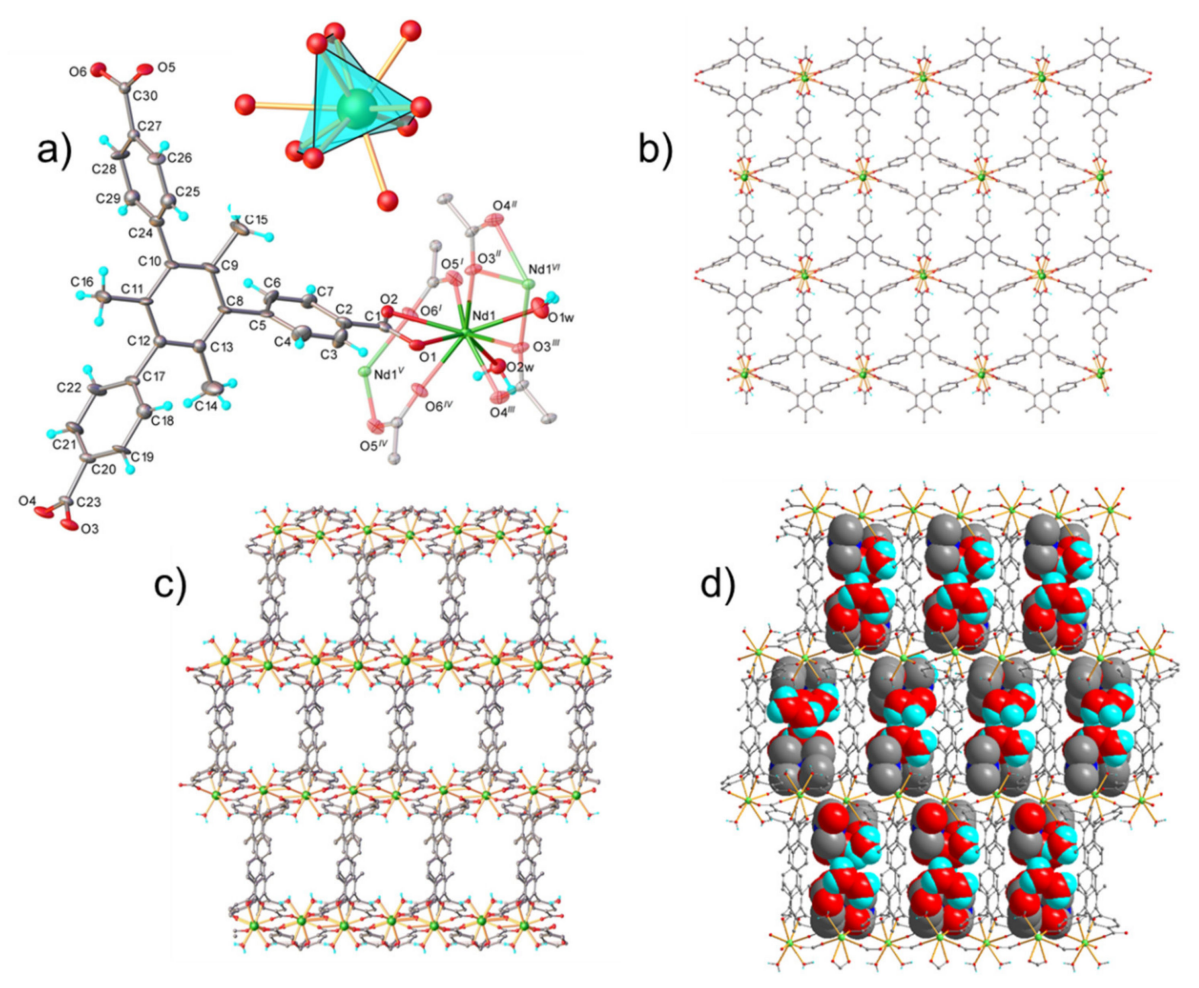
Crystal structure of [NdL(H_2_O)_2_]⋅1.33DMF⋅2H_2_O (**3**). Extended view of the asymmetric part and coordination environment around the Nd atom in **3** along with the atom labeling scheme and thermal ellipsoids at the 40% probability level. Symmetry generated fragments are drawn with 50% transparency (**a**). Crystal structure packing viewed along the *a* axis (**b**). View of the crystal structure showing the formation of the channels along the *b* axis (**c**); with space filling representation of the co-crystallized DMF and H_2_O solvent molecules (**d**). Symmetry codes: I = 0.5 + *x*, 1.5 − *y*, 0.5 + *z*; II = 0.5 − *x*, 0.5 + *y*, 1.5 − *z*; III 0.5 + *x*, 0.5 − *y*, 0.5 + *z*; IV = 0.5 − *x*, −0.5 + *y*, 1.5 − *z*; V = 1 − *x*, 1 − *y*, 2 − *z*; VI −*x*, 1 − *y*, 2 − *z*.

The results of single-crystal x-ray diffraction studies are shown in Figure 2; Figure 3. It should be noted that the three complexes described here exhibited similar structural features, being constructed in the same way. Moreover, compounds **1** and **2** were isostructural, therefore only the structure of compound [LaL(H_2_O)_2_]_n_ (**1**) is described below in detail (Figure 2). Complex **1** crystallizes in the orthorhombic space group Pnna. As shown in Figure 2a, the asymmetric unit comprises a La atom in a special position on a two-fold axis, half of the deprotonated L^3−^ ligand, and one coordinated water molecule. The L^3−^ tricarboxylate linker acts as an eight-dentate ligand, bridging and chelating toward five La atoms. Two of the carboxylate groups are coordinated in μ_2_-*к^3^O,O’:O* tridentate-bridging mode, while the third group exhibits a *к^2^O,O*’ bidentate-chelating function. The La atom is coordinated by ten oxygen atoms in a distorted square antiprism geometry (Figure 2a polyhedron). The La–O bond distances vary in the range 2.503(7)−3.017 Å and 2.461(4)−3.177(4) Å for [LaL(H_2_O)_2_]_n_ (**1**) and [CeL(H_2_O)_2_]_n_ (**2**), respectively. In the crystal, the asymmetric units are assembled to form a three-dimensional coordination network (Figure 2b–d). It can be described as formed by infinite linear arrays of La atoms bridged by μ_2_-*к^3^O,O’:O* tridentate-bridging/chelating carboxylate groups along the *a* axis. These linear chains are further interconnected via tripodal spacers L^3−^. Within the linear chain, the two neighboring La atoms are separated at 4.690(4) Å across the tricarboxylate spacers at 16.356(4) Å. The network structure forms large rectangular channels of 17.5 × 12.7 Å (taking into account the van-der-Waals surfaces) directed along the *b* axis (Figure 2c), indicating the potential porosity of this compound. The solvent-accessible volume of the channels is equal to 2060 Å^3^/unit cell, which constitutes 48% of the unit cell volume. 

Compared with compounds **1** and **2**, the x-ray diffraction study for **3** yielded very similar unit cell dimensions. Nevertheless, compound **3** crystallized in the monoclinic space group P2_1_/n, having a *β* angle of 93.098(18)°. The crystal structure is formed by the [NdL(H_2_O)_2_]_n_ coordination polymer and solvate molecules of H_2_O and DMF in a 1:2:1.33 stoichiometric ratio. A view of the asymmetric unit is shown in Figure 3a. The coordination environment of Nd comprises nine oxygen atoms, seven of which come from five carboxylate groups and two from coordinated water molecules. The coordination polyhedron was different compared to compounds **1** and **2**: this can be characterized as a tricapped trigonal prism (Figure 3a polyhedron). The Nd–O bond distances vary in the range of 2.464(6)−2.947(6) Å. As shown in Figure 3, the three-dimensional network in **3** closely resembles that found for **1** and **2** (cf. Figure 2b,c). In the structure of **3**, the positions of the H_2_O and DMF solvent molecules could be easily determined from Fourier maps and refined. A view of the crystal structure along the *b* axis showing the channels accommodating the solvent molecules is depicted in Figure 3d.

### 2.2. Infrared Spectroscopy Attenuated Total Reflectance (ATR) 

For all synthesized lanthanide metal-organic frameworks, the ATR spectra were quite similar and are given in the Supplementary Materials (Figures S2–S9). The IR spectra showed strong characteristic adsorptions in the 1610–1520 cm^−1^ and 1420–1370 cm^−1^ interval, assigned to the symmetric/asymmetric COO^−^ vibrations of the ligand carboxylate groups. The broad bands between 3600 and 3200 cm^−1^ can be attributed to the hydrogen bonded υOH groups from adsorbed water. 

### 2.3. Powder X-ray Diffraction

Powder X-ray diffraction (PXRD) reveals information about the crystallinity and crystalline phase purity of the material. The PXRD patterns were recorded after the compounds had been filtered, air-dried, and exposed to air for at least a few days (Figures S10–S20). The patterns recorded for all compounds were in good agreement with each other, as can be seen in Figure S20. The simulation versus experimental patterns for **1**–**3** are represented in Figures S11–S13. 

### 2.4. Thermogravimetric Analysis (TG/DTG) 

Thermal stability and the pathways of thermal decomposition of all complexes were investigated by means of the TG-DTG (nitrogen) methods (see Figures S21–S28). The newly synthesized Ln-MOFs were found to have high thermal stability compared with the ligand. For compounds **1** and **2**, the first weight loss of 3–4% corresponded to the loss of water molecules. Above around 480 °C, the decomposition process of the framework and organic ligand takes place. The TG trace of the as-synthesized **3** showed a mass loss of ~11% up to 220 °C, which can be attributed to the loss of water molecules and part of the DMF molecules (theor. ~21% for water and DMF). When compound **3** was analyzed after activation and gas sorption measurements, no mass loss up to 480 °C was observed, which is evidence of successful solvent removal. During heating of **5**, the desolvation process took place in three steps. The first weight loss (~5%) found in the temperature range of 40–220 °C is associated with the loss of solvent molecules (calc. 4.5%). Further heating caused the decomposition of the organic ligand. The thermal decomposition curves of compounds **4**, **6**, and **7** showed a similar behavior and high thermal stability at about 500 °C, as presented in the SM. It is worth pointing out that the MOFs presented here have a high thermal stability that is similar to other lanthanide metal organic frameworks [42].

### 2.5. Sorption Properties

In order to assess the porous nature of compounds **1**–**7**, the nitrogen adsorption–desorption isotherms at −196 °C were recorded. Prior to measurement, the samples were outgassed for 4 h under a high vacuum (2.7 × 10^−2^ mbar) at 80 °C. The compounds exhibited permanent porosity with BET surface areas from 110 to 470 cm^2^/g, and total pore volumes between 0.1 to 0.2 cm^3^/g (Table 1). The obtained nitrogen isotherms are shown in Figures S30–S36 in the Supplementary Materials. 

**Figure 4:**
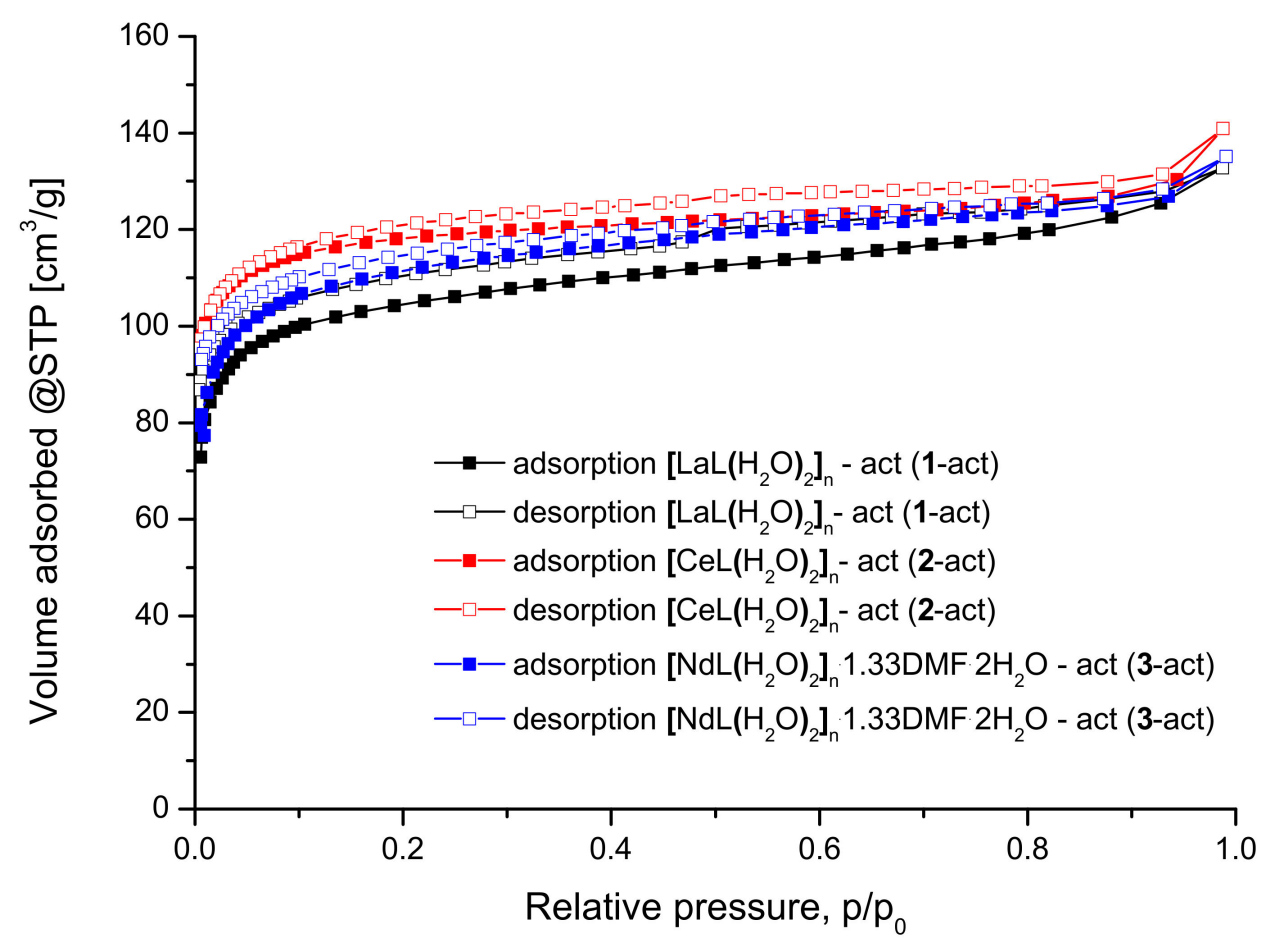
N_2_ sorption isotherms of the activated compounds of **1**–**3** at 77 K. Closed and open symbols refer to adsorption and desorption, respectively.

The structurally authenticated compounds **1**–**3** exhibited adsorption isotherm branches as a composite of Types I and II with a H4 hysteresis loop. The pronounced uptake at low p/p_0_ is associated with the filling of micropores. H4 loops are often found with aggregated crystals or micro-mesoporous materials [43,44]. Of note, structurally similar compounds **1**–**3** (see Figure 4) also exhibited rather similar surface areas between 400–460 m^2^/g and a total pore volume of 0.19–0.20 cm^3^/g. In a repeated adsorption/desorption cycle, the surface area remains unchanged within the experimental error [44]. 

Compounds **4**–**7** still feature adsorption isotherm branches that can be viewed as a composite of Types I and II. However, there is essentially no desorption until p/p_0_ <~0.1–0.15 for **4**–**6** (for **6** after the second adsorption run). For **7**, the desorption starts around p/p_0_ <~0.3. For **4**–**7**, about 80% of the adsorbed N_2_ is still retained at a very low p/p_0_ of ~0.01. There is a final steep desorption branch at p/p_0_ of ~0.01 for these remaining 80%. Such a very steep desorption branch is also a characteristic feature of H2(a) hysteresis loops and can be attributed either to pore-blocking/percolation in a narrow range of pore necks or to cavitation-induced evaporation [44].

A similar sorption behavior with broad isotherms and steep desorption steps at low pressures was frequently observed for H_2_ and CH_4_ adsorption in IFP MOFs [45,46,47,48] and in supramolecular hydrogen-bonded imidazolate frameworks (HIFs) [49,50] with gate effects (IFP = imidazolate framework Potsdam), in part also for CO_2_ adsorption in IFPs [47,51]. Such a broad desorption behavior is rarely observed in microporous MOFs and only observed for MOFs with flexible substituents. The structurally authenticated IFPs possess very small, less than 1 Å, pore aperture windows to the cavities and flexible ethoxy or methoxy groups form the pore aperture windows [47,48]. For H_2_ sorption at 77 K IFP-7 [48], IFP-8 [45,46] and IFP-9 [48] exhibits an open-loop hysteresis due to almost irreversible adsorption at higher relative pressure. Although H_2_ is a small molecule with a kinetic diameter of only 2.89 Å at 77 K, the sorption isotherms can deviate from ideal equilibrium experiments as pronounced kinetic effects occur because of the small channel size and the gate effect. In IFP-7, 98% of the adsorbed H_2_ is trapped in the framework when the pressure is reduced from 840 mmHg to 100 mmHg, and 70% of the adsorbed H_2_ remains when the pressure is further reduced to 7 mmHg [48]. In IFP-8, 98% of the adsorbed H_2_ is trapped in the framework when the pressure is reduced from 760 mmHg (1 bar) to 114 mmHg (0.15 bar) [45]. 

### 2.6. Luminescence

**Figure 5:**
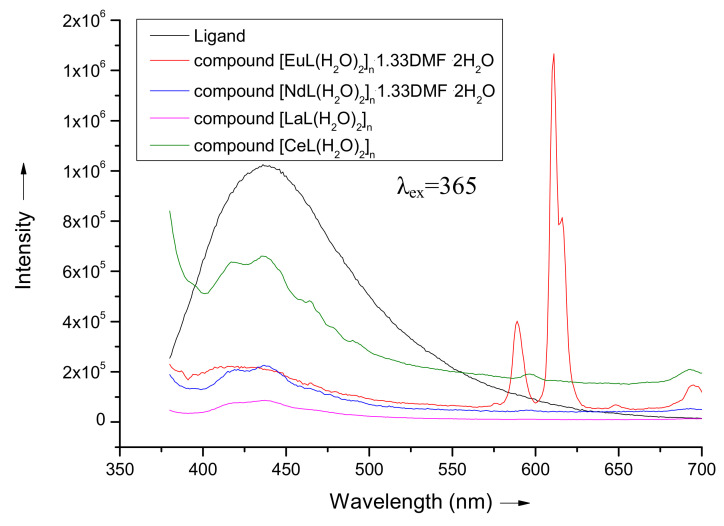
Emission spectra of ligand and lanthanide MOF **1**–**4** obtained under excitation at 365 nm.

It is known that lanthanides often exhibit luminescence properties [1,16,52]. In order to study the luminescence properties of the synthesized frameworks, the fluorescence spectra of Ln-MOFs (**1**, **2**, **3,** and **4**) as well as ligand H_3_L were recorded in solid state (powder) at room temperature. It was found that H_3_L displays an emission maximum at 437 nm upon excitation at λ = 365 nm. Similar emissions in the UV region were observed for frameworks **1**, **2**, and **3** (Figure 5), which can be attributed to the organic ligand. In the case of the Eu based framework **4**, besides the weak blue emission of the ligand, typical red emissions attributed to the Eu^3+^ ion were observed. The characteristic sharp emissions at λ = 561 nm (very weak, ^5^D_0_→^7^F_0_), λ = 589 nm (medium, ^5^D_0_→^7^F_1_), λ = 611 nm (very strong, ^5^D_0_→^7^F_2_), λ = 616 nm (very weak, ^5^D_0_→^7^F_3_), and λ = 697 nm (very weak, ^5^D_0_→^7^F_4_) were in good correlation with the data reported in the literature [53,54,55]. The red emissions of framework **4** were confirmed through fluorescence microscopy by irradiating at λ = 365 nm, as can be seen in the SM, Figure S37.

### 2.7. Catalytic Activity

Two Ln-MOFs (**1** and **3**) were investigated in O-acylation reactions to explore their Lewis-acidic properties. Despite the large number of reported O-, N-, S- acylation processes [56,57], in the last decade, the involvement of MOFs in heterogeneous catalysis has greatly increased [58,59]. The advantages of MOF materials are solid-state catalytic activity, together with the robustness of their structure, allowing facile separation after the catalytic process [56,59,60]. 

**Scheme 1:**
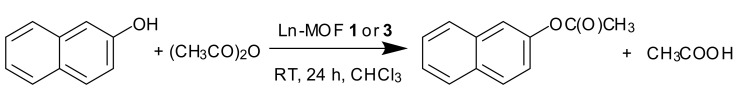
Acylation of 2–naphthol with acetic anhydride in the presence of **1** or **3** as the catalyst.

In the present work, the conversion of 2-naphthol to 2-naphthyl acetate by the reaction with (CH_3_COO)_2_O in the presence of lanthanide-MOFs as catalysts (Scheme 1) was investigated. The reactions were carried out with 1.5 equivalents of acetic anhydride at room temperature in the presence of 1 mol% of catalysts and chloroform as the solvent for 24 h.

In the control experiment, in the absence of the catalyst, the yield of the acylated product was lower than 10%. Before each catalytic experiment, the catalyst was activated by washing several times with chloroform and then drying in an oven at 80 °C for 20 h. The catalytic activity of the lanthanide-based MOFs was tested in multiple run experiments (see Table 2). When the reactions were catalyzed by **1** (1 mol%), the yield was 88%. When the catalyst used was **3**, the yields were 98% (run 1), 96% (run 2), and 92% (run 3) (see Table 2, Entry 1–4). The spectral data of the isolated yellowish powders of 2-naphthylacetate were in full agreement with the authentic samples (Mp, NMR, see Figure S29, SM) [61]. The results were expressed as the average of at least two independent measurements performed for each sample.

Critically important for MOF catalysts is the framework stability during catalysis. For compound **3**, this was tested over three runs and the results showed a stable catalytic activity. In addition, after the first and the third run, the catalyst was analyzed for crystallinity by PXRD. The results indicate that the compound was only stable over one run, but had significantly deteriorated after the third run (Figure S15, SM). Moreover, two catalytic reactions were carried out with the activated and non-activated catalyst. It is clear that without activating the catalyst, the yield is much lower (see Table 2, entry 6).

In order to prove the heterogeneity of the catalytic process, a room temperature filtration test was performed. The catalyst was removed from the reaction mixture after 5 h using a glass frit funnel. The results show that after removing the catalyst, the reaction did not proceed further, indicating that no catalytically active materials remained in the filtrate (Table 2, entry 5).

## 3. Material and Methods

All chemicals were purchased from commercial suppliers and used as received. The Nuclear Magnetic Resonance (NMR) spectra were recorded on a BRUKER AVANCE III 400 (Bruker BioSpin Rheinstetten, Germany) instrument operating at 400.1 and 100.6 MHz for ^1^H and ^13^C nuclei, respectively. Carbon, Hydrogen, Nitrogen elemental analysis was performed on a vario Micro cube CHNS analyzer from Elementar Analysensysteme GmbH, Germany. Infrared (IR) spectra were recorded on a BRUKER VERTEX 70 (Bruker Optik GmbH, Ettlingen, Germany) FTIR spectrometer with the measurements performed in ATR (attenuated total reflectance) Golden Gate^®^ mode in the 600–4000 cm^−1^ range at room temperature with a resolution of 4 cm^−1^ and accumulation of 64 scans. Powder x-ray diffraction (PXRD) patterns were collected on a Bruker AXS D2 Phaser powder diffractometer (Bruker AXS GmbH, Karlsruhe, Germany) equipped with a flat silicon, low background sample holder using Cu-Kα radiation (λ = 1.5418 Å) with a scan speed of 0.2 s/step and a step size of 0.02° (2θ) at 290 K in the range of 2θ = 5–40°. Simulated PXRD patterns were calculated with the CCDC Mercury 3.1 program (Cambridge, UK) using the single-crystal data of the compounds. A STA 449F1 JUPITER (Netzsch, GmbH, Selb, Germany) thermal analyzer from Netzsch was employed for the thermogravimetric (TG) measurements at a heating rate of 5 °C min^−1^ between 30 and 700 °C. The data were processed with the NETZSCH PROTEUS 4.2 software (Netzsch, GmbH, Selb, Germany). Nitrogen sorption experiments (up to 1 bar) for the BET surface area and porosity determination were measured with a Quantachrome NOVA 4200e (Quantachrome GmbH & Co. KG, Odelzhausen, Germany) at 77 K. About 20 to 40 mg of freshly synthesized samples were weighed before and after the degassing procedure to confirm the evacuation of the solvent. The fluorescence microscopy imaging was performed on a Leica DMI 3000 B (Leica Microsystems, Germany) microscope equipped with “A” and “GFP” filter cubes, with UV (365 nm) and green excitation (470 nm), respectively. The solid-state fluorescent study was conducted at room temperature on a Horiba Fluoromax-4 fluorescence spectroscopy instrument (HORIBA Jobin Yvon GmbH, Bensheim, Germany).

### 3.1. Synthesis of the Ligand 1,3,5-tris(4-carboxyphenyl)-2,4,6-trimethylbenzene (H_3_L)

The linker H_3_L was prepared according to a procedure described in the literature [30] and its structure and purity were confirmed by NMR and IR spectroscopy. 

^1^H NMR (DMSO-d6, 400.1 MHz, ppm): δ = 12.9 (s, 3H); 8.04 (d, ^3^J_H,H_ = 8.04 Hz, 6H); 7.35 (d, ^3^J_H,H_ = 8.04 Hz, 6H); 1.62 (s, 9H). ^13^C NMR (DMSO-d6, 100.6 MHz, ppm): δ = 167.6; 146.3; 139.2; 132.3; 130.1; 129.9; 129.8; 19.6. IR (ATR): ν (cm^−1^) = 640.33 (m); 661.54 (w); 682.76 (w); 715.50 (m); 759.91 (s); 796.55 (m); 864.06 (s); 960.49 (w); 1018.35 (m): 1043.42 (w); 1060.78 (w); 1097.43 (m); 1143.72 (vw); 1178.43 (w); 1253.65 (w); 1278.73 (w); 1305.73 (m); 1411.81 (vs); 1519.81 (s); 1583.46 (vs); 1664.47 (s); 1820.69 (vw); 1944.13 (vw); 2279.72 (vw); 2561.31 (vw); 2750.32 (vw); 2923.90 (w); 3355.93 (w); 3512.16 (w); 3807.25 (vw).

### 3.2. Typical Synthesis of Ln-MOFs Where Ln = La (**1**), Ce (**2**), Nd (**3**), Eu (**4**), Gd (**5**), Dy (**6**), Ho (**7**)

All complexes were prepared in the same manner using the following procedure. To a solution of H_3_L (0.01 g, 0.02 mmol) in dimethylformamide (DMF) (except La(NO_3_)_3_∙6H_2_O and Ce(NO_3_)_3_∙6H_2_O where ethanol was used) in a 20 mL culture tube was added Ln(NO_3_)_3_∙6H_2_O (~0.036 g, 0.08 mmol) dissolved in ethanol/water at room temperature (see Table S1, Supplementary Materials (SM) for the volumes and solvents used). The tube with a clear solution was kept under static conditions for 1–5 days at 80 °C (reaction times are detailed in Table S1, SM). After cooling, the crystalline product was collected by centrifugation and washed with DMF and ethanol. Finally, the crystals were dried at room temperature. Specific conditions for each Ln-MOF are given in section S1, SM.

[LaL(H_2_O)_2_]_n_ (**1**): Yield 62%; Elemental analysis calcd. (%) for C_30_H_25_LaO_8_ (*M* = 652.41 g/mol): C 55.26, H 3.86; found: C 55.26, H 3.79. IR (ATR): ν (cm^−1^) = 638.41 (m); 675.06 (w); 717.49 (m); 758.01 (vs); 858.29 (s); 958.58 (m); 1062.74 (w); 1105.17 (w); 1141.82 (m); 1176.53 (m); 1236.32 (m); 1284.54 (m); 1307.69 (m); 1375.29 (vs); 1529.85 (s); 1585.43 (s); 1602.78 (s); 2929.76 (w); 2987.62 (w); 3620.25 (w).

[CeL(H_2_O)_2_]_n_ (**2**): Yield 88%; Elemental analysis calcd. (%) for C_30_H_25_CeO_8_ (*M* = 653.63 g/mol): C 55.13; H 3.86 found C 55.83, H 3.92. IR (ATR): ν (cm^−1^) = 638.42 (w); 715.57 (m); 758.01 (s); 796.58 (m); 862.16 (m); 958.60 (w); 1016.46 (m); 1037.68 (w); 1101.33 (w); 1139.90 (w); 1176.55 (m); 1276.85 (m); 1305.78 (m); 1406.07 (vs); 1523.73 (s); 1583.52 (s); 1608.59 (s); 2922.09 (w); 3429.35 (w); 3616.44 (w). 

[NdL(H_2_O)_2_]⋅1.33DMF⋅2H_2_O (**3**): Yield: 84%; Elemental analysis calcd. (%) for C_34_H_38.3_O_11.33_N_1.33_Nd (*M* = 791.21 g/mol): C 51.59, H 4.87; N 2.35 found: C 52.30, H 4.57, N 2.56. IR (ATR): ν (cm^−1^) = 638.41 (m); 675.06 (w); 715.76 (m); 758.01(s); 796.57 (m); 862.15 (s); 958.58 (m); 1016.45 (m); 1101.31 (m); 1139.89 (w); 1176.53 (m); 1267.53 (m); 1305.76 (m); 1382.91 (vs); 1402.2 (vs); 1523.71 (s); 1581.57 (s); 1604.71 (s); 2923.37 (w); 3627.96 (w). 

[EuL(H_2_O)_2_]⋅1.33DMF⋅2H_2_O (**4**): Yield: 66%; Elemental analysis calcd. (%) for C_34_H_38.3_O_11.33_N_1.33_Eu (*M* = 797.09 g/mol): C 51.08, H 4.83, N 2.33 (calc idem Nd), found C 51.45, H 5.01; N 2.50. IR (ATR): ν (cm^−1^) = 640.34 (m); 715.56 (s); 758 (vs); 790.79 (w); 864.08 (s); 958.58 (m); 1022.53 (m); 1203.24 (m); 1141.82 (m); 1176.53 (m); 1240.18 (m); 1280.68 (m); 1307.69 (m); 1377.12 (vs); 1402.02 (vs); 1519.85 (s); 1583.5 (s); 1583.5 (s); 1606.71 (s); 2927.83 (w); 2981.83 (w); 3649.18 (w).

[GdL(H_2_O)_2_]⋅2DMF⋅2H_2_O (**5**): Yield: 82%; Elemental analysis calcd. (%) for C_36_H_43_N_2_O_12_Gd (*M* = 852.98 g/mol): C 50.69, H 5.08, N 3.28, found C 50.87, H 4.75; N 3.85. IR (ATR): ν (cm^−1^) = 640.33 (m); 675.04 (w); 715.55 (s); 757.98 (vs); 794.62 (m); 864.06 (s); 958.56 (m); 1016.42 (m); 1103.21 (m); 1143.72 (m); 1176.50 (m); 1245.94 (m); 1278.73 (m); 1303.80 (m); 1384.80 (vs); 1406.02 (vs); 1514.03 (s); 1527.53 (s); 1606.67 (s); 1934.748(w); 2923.63 (w); 3039.63 (w); 3406.08 (w); 3616.31 (w); 3652.95 (w). 

[DyL(H_2_O)_2_]_n_ (**6**): Yield: 77%; Elemental analysis calcd. (%) for C_30_H_25_O_8_Dy (*M* = 677.08 g/mol): C 53.30, H 3.73; found: C 53.81, H 4.08. IR (ATR): ν (cm^−1^) = 639.33 (m); 715.55 (m); 757.98 (s); 794.65 (m); 865.98 (s); 958.56 (w); 1016.42 (m); 1101.21 (m); 1141.72 (m); 1176.50 (m); 1278.72 (m); 1377.09 (vs); 1583.46 (s); 1936.48 (w); 2854.44 (w); 2921.9 (w); 3419.65 (w); 3508.37 (m). 

[HoL(H_2_O)_2_]⋅1.33DMF⋅2H_2_O (**7**): Yield 45%; Elemental analysis calcd. (%) for C_34_H_38.3_O_11.33_N_1.33_Ho (*M* = 810.06 g/mol): C 50.26, H 4.76, N 2.22, found: C 50.73, H 5.06., N 2.90. IR (ATR): ν (cm^−1^) = 640.33 (m); 715.55 (s); 757.98 (vs); 794.65 (m); 865.98 (s); 958.56 (w); 1016.42 (m); 1016.21 (m) 1103.21 (m); 1143.72 (m); 1176.50 (m); 1278.72 (m); 1305.73 (m);1376.02 (vs) 1527.53 (s); 1583.46 (vs); 1814.9 (vw); 1936.48 (w); 2281.65 (vw); 2559.38 (vw); 2856.40 (w); 2923.9 (w); 3425.37 (w); 3514.09 (w). 

### 3.3. Synthesis of 2-Naphthyl Acetate

A mixture of the substrate, 2–naphthol (0.07 g, 0.48 mmol), acetic anhydride (0.07 g, 0.72 mmol, 1.5 equiv.), and Ln-MOFs as the catalyst (1 mol%—related to substrate) was stirred at room temperature with CHCl_3_ (2 mL) as the solvent for one day. The catalyst was collected by filtration and the organic phase was washed successively with 2% aqueous NaOH (5 mL) and saturated NaCl solution (5 mL). After drying with MgSO_4_, filtration and evaporation of the solvent at reduced pressure, the solid product was isolated and analyzed (yellowish powder; Mp: 72 °C—lit. 71 °C [61]). Yield 98% (**3** as catalyst). ^1^H NMR (CDCl_3_-d_3_, 400.1 MHz, ppm): δ = 7.89–7.86 (m, 2CH); 7.84–7.82 (m, CH; ^3^J_H,H_ = 7.9 Hz); 7.61 (d, CH; ^3^J_H,H_ = 2.3 Hz); 7.51 (m, 2CH); 7.27 (dd, CH; ^3^J_H,H_ = 9.03 Hz); 2.38 (s, CH_3_). ^13^C NMR (CDCl_3_-d, 100.6 MHz, ppm); δ = 169.7; 148.3; 133.7; 131.4; 129.3; 127.7; 127.6; 126.5; 125.7; 121.1; 118.5; 21.2.

### 3.4. Crystal Structure Determination 

X-ray diffraction measurements were carried out with an Oxford-Diffraction Xcalibur E CCD diffractometer equipped with graphite-monochromated MoKα radiation. Single crystals were positioned at 40 mm from the detector and 163, 157, and 257 frames were measured each for 70, 10, and 75 s over a 1° scan width for **1**, **2**, and **3**, respectively. The unit cell determination and data integration were carried out using the CrysAlis package of Oxford Diffraction [62]. The structures were solved by direct methods using Olex2 [63] and refined by full-matrix least-squares on *F*^2^ with SHELXL-2015 [64] using an anisotropic model for non-hydrogen atoms. The positional parameters for H atoms attached to C atoms were introduced in idealized positions (d_CH_ = 0.96 Å) using the riding model with their isotropic displacement parameters fixed at 120% of their riding atom. Positional parameters of the H attached to O atoms were obtained from different Fourier syntheses and verified by the geometric parameters of the corresponding hydrogen bonds. Crystal structures **1** and **2** contained large areas of disordered solvent molecules that could not be localized. In these cases, the contribution of disordered solvent molecules to structure factors was extracted using the “Solvent Mask” tool available in Olex2. Crystals of **3** were found to be a non-merohedral twin with two components related by 180° rotation about the [100] direct lattice direction. The twin component ratio was refined at 0.52:0.48. The molecular plots were obtained using the Olex2 program. The crystallographic data and refinement details are quoted in Table 3, while bond lengths are summarized in Tables S2–S4 (Supplementary Materials). CCDC-1904154 (**1**), CCDC-1904155 (**2**), and CCDC-1904156 (**3**) contain the supplementary crystallographic data for this contribution. These data can be obtained free of charge via www.ccdc.cam.ac.uk/conts/retrieving.html (or from the Cambridge Crystallographic Data Center, 12 Union Road, Cambridge CB2 1EZ, UK; fax: (+44) 1223-336-033; or deposit@ccdc.ca.ac.uk).

## 4. Conclusions 

A series of new lanthanide metal-organic frameworks with the general formula [LnL(H_2_O)_2_]_n_ (where Ln = La, Ce, Nd, Eu, Gd, Dy and Ho, and L = deprotonated *1,3,5*-tris(*4*-carboxyphenyl)-*2,4,6*-trimethylbenzene) were synthesized. The crystal structures of the La(III), Ce(III), and Nd(III) complexes were successfully studied by the single-crystal diffraction method. It was demonstrated that all of these compounds showed high thermal stability (up to ~480 °C), while the gas sorption studies indicated good permanent porosity with SBET up to ~400 m^2^/s. The catalytic activity for a model reaction, namely *O*-acetylation was also evaluated and it was found that the synthesized MOFs successfully catalyzed the acetylation of 2-napthol. The novel [LnL(H_2_O)_2_]_n_ MOFs can serve as promising materials for gas storage as well as for heterogeneous catalysis.

## Figures and Tables

**Table 1:** BET surface area and total pore volume for **Ln-MOFs** compound.

Ln-MOFs—Activated Compounds	BET Surface Area [m^2^/g]	Total Pore Volume [cm^3^/g]
[LaL(H_2_O)_2_]_n—_act (**1-act**)	405/395 *	0.19
[CeL(H_2_O)_2_]_n—_act (**2-act**)	467/460 *	0.20
[NdL(H_2_O)_2_]_n—_act (**3-act**)	426/404 *	0.20
[EuL(H_2_O)_2_]_n—_act (**4-act**)	114	0.08
[GdL(H_2_O)_2_]_n—_act (**5-act**)	348	0.18
[DyL(H_2_O)_2_]_n—_act (6-act) [DyL(H_2_O)_2_]_n—_act (**6-act**)	202 (Figure S35a) 298 (Figure S35b)	0.16 0.16
[HoL(H_2_O)_2_]_n—_act (**7-act**)	286	0.15

* The same compound, second measuring, without exposing to air. The experimental error for a BET surface area is about 5–10%.

**Table 2:** Acylation of O–substrates with Ac_2_O in the presence of **1** and **3** as catalysts.

Entry	Substrate	Conversion *	Time	Catalyst	Run Number
1	2-naphthol	88	24	**1**	1
2	2-naphthol	98	24	**3**	1
3	2-naphthol	96	24	**3**	2
4	2-naphthol	92	24	**3**	3
5	2-naphthol	65 65	5 24	**3** -	1 2
6 **	2-naphthol	80 72	24 24	**3** **3**	1 2

* Every experiment was performed at least in duplicate; ** The catalyst is dried in air.

**Table 3:** Crystal data and details of the data collection for **1**–**3**.

Compound	3984 (1)	3971 (2)	3972 (3)
empirical formula	C_30_H_25_LaO_8_	C_30_H_25_CeO_8_	C_34_H_38.33_N_1.33_NdO_11.33_
*Fw*	652.41	653.62	791.21
space group	*Pnna*	*Pnna*	*P*2_1_/*n*
*a* [Å]	9.3345(5)	9.5029(6)	9.5200(13)
*b* [Å]	16.6700(9)	16.6504(10)	16.473(3)
*c* [Å]	27.2304(19)	27.1399(15)	27.389(6)
*a* [°]	90	90	90
*B* [°]	90	90	93.098(18)
*g* [°]	90	90	90
*V* [Å^3^]	4237.2(4)	4294.3(4)	4288.9(13)
*Z*	4	4	4
*r*_calcd_ [g cm^−3^]	1.023	1.011	1.225
Crystal size [mm]	0.20 × 0.20 × 0.10	0.40 × 0.20 × 0.20	0.70 × 0.60 × 0.18
*T* [K]	180	293	180
*μ* [mm^−1^]	1.040	1.092	1.260
2È range	5.11 to 50.054	4.542 to 50.052	2.886 to 50.054
Reflections collected	9929	9514	11840
Independent reflections	3744 [*R*_int_ = 0.0515]	3780 [*R*_int_ = 0.0952]	11840 [*R*_int_ = 0.1178]
Data/restraints/parameters	3744/54/181	3780/12/180	11840/48/439
*R*_1_^[a]^	0.0852	0.0538	0.0651
*wR*_2_^[b]^	0.2280	0.0983	0.1431
GOF ^[c]^	1.048	1.002	1.040
Largest diff. peak/hole/e Å^−3^	1.16/−1.45	1.23/−1.58	1.40/−1.00

^[a]^*R*_1_ = Σ||*F*_o_| − |*F*_c_||/Σ|*F*_o_|. ^[b]^ *wR*_2_ = {Σ[*w*(*F*_o_^2^ − *F*_c_^2^)^2^]/Σ[*w*(*F*_o_^2^)^2^]}^1/2^. ^[c]^ GOF = {Σ[*w*(*F*_o_^2^ − *F*_c_^2^)^2^]/(*n* − *p*)}^1/2^, where *n* is the number of reflections and *p* is the total number of parameters refined.

## Supplementary Materials

The following are available online, Synthesis of the ligand *1,3,5*-tris(4-carboxyphenyl)-*2,4,6*-trimethylbenzene (H_3_L) and of Ln-MOFs compounds (**1**–**7**). Table S1: Overview of the experimental details for the synthesis of **1**–**7**. Figure S1: Optical microscopy image of **1**–**7**, with Leica ICC50 W, 4x/0.10. Figure S2: IR spectrum of ligand H_3_L. Figure S3: IR spectrum of compound [LaL(H_2_O)_2_]_n_ (**1**). Figure S4: IR spectrum of compound [CeL(H_2_O)_2_]_n_ (**2**). Figure S5: IR spectrum of compound [NdL(H_2_O)_2_]_n_·1.33DMF·2H_2_O (**3**). Figure S6: IR spectrum of compound [EuL(H_2_O)_2_]_n_·1.33DMF·2H_2_O (**4**). Figure S7: IR spectrum of compound [GdL(H_2_O)_2_]_n_·2DMF·2H_2_O (**5**). Figure S8: IR spectrum of compound [DyL(H_2_O)_2_]_n_ (**6**). Figure S9: IR spectrum of compound [HoL(H_2_O)_2_]_n_·1.33DMF·2H_2_O (**7**). Figure S10: PXRD patterns of the two isostructural compounds [LaL(H_2_O)_2_]_n_ (**1**) and [CeL(H_2_O)_2_]_n_ (**2**) recorded at different times (6 min and 30 min). Figure S11: PXRD patterns of [LaL(H_2_O)_2_]_n_ (**1**) recorded at different times (6 min and 30 min). Figure S12: PXRD patterns of [LaL(H_2_O)_2_]_n_ (**2**). Figure S13: PXRD patterns of [NdL(H_2_O)_2_]_n_·1.33DMF·2H_2_O (**3**). Figure S14: PXRD patterns of compound [NdL(H_2_O)_2_]_n_·1.33DMF·2H_2_O (**3**)-red, compound **3** after basic condition-black; compound **3** after acidic condition-green. Figure S15: PXRD patterns of activated compound [NdL(H_2_O)_2_]_n_·1.33DMF·2H_2_O (**3**)-black and compound (**3**) after catalytic reaction (first cycle) -red and (**3**) -blue, after third cycle in catalytic processes. Figure S16: PXRD patterns of [EuL(H_2_O)_2_]_n_·1.33DMF·2H_2_O (**4**). Figure S17: PXRD patterns of [GdL(H_2_O)_2_]_n_·2DMF·2H_2_O (**5**). Figure S18: PXRD patterns of [DyL(H_2_O)_2_]_n_ (**6**). Figure S19: PXRD patterns of [HoL(H_2_O)_2_]_n_·1.33DMF·2H_2_O (**7**). Figure S20: PXRD patterns of all compounds (**1**–**7**). Figure S21: Thermogravimetric analysis of ligand H_3_L. Figure S22: Thermogravimetric analysis of compound [LaL(H_2_O)_2_]_n_ (**1**). Figure S23: Thermogravimetric analysis of compound [CeL(H_2_O)_2_]_n_ (**2**). Figure S24: Thermogravimetric analysis of compound [NdL(H_2_O)_2_]_n_·1.33DMF·2H_2_O (**3**). Figure S25: Thermogravimetric analysis of compound [EuL(H_2_O)_2_]_n_·1.33DMF·2H_2_O (**4**). Figure S26: Thermogravimetric analysis of compound [GdL(H_2_O)_2_]_n_·2DMF·2H_2_O (**5**). Figure S27: Thermogravimetric analysis of compound [DyL(H_2_O)_2_]_n_·(**6**). Figure S28: Thermogravimetric analysis of compound [HoL(H_2_O)_2_]_n_·1.33DMF·2H_2_O (**7**). Figure S29: Thermogravimetric analysis of 2-naphyl acetate. Figure S30: Nitrogen sorption isotherms of [LaL(H_2_O)_2_]_n_ (**1**) recorded at 77 K. Filled symbols are for adsorption, empty symbols are for desorption (SBET = 405 m^2^/g). Figure S31: Nitrogen sorption isotherms of [CeL(H_2_O)_2_]_n_ (**2**) recorded at 77 K. Filled symbols are for adsorption, empty symbols are for desorption (SBET = 467 m^2^/g). Figure S32: Nitrogen sorption isotherms of [NdL(H_2_O)_2_]_n_·1.33DMF·2H_2_O (**3**) recorded at 77 K. Filled symbols are for adsorption, empty symbols are for desorption (SBET = 426 m^2^/g). Figure S33: Nitrogen sorption isotherms of [EuL(H_2_O)_2_]_n_·1.33DMF·2H_2_O (**4**). Filled symbols are for adsorption, empty symbols are for desorption (SBET = 114 m^2^/g). Figure S34: Nitrogen sorption isotherms of [GdL(H_2_O)_2_]_n_·2DMF·2H_2_O (**5**) recorded at 77 K. Filled symbols are for adsorption, empty symbols are for desorption (SBET = 348 m^2^/g). Figure S35: (a) Nitrogen sorption isotherms of [DyL(H_2_O)_2_]_n_ (**6**). Filled symbols are for adsorption, empty symbols are for desorption (SBET = 202 m^2^/g), (b) Nitrogen sorption isotherms of [DyL(H_2_O)_2_]_n_ (**6**) (different synthesis, the same conditions). Filled symbols are for adsorption, empty symbols are for desorption (SBET = 298 m^2^/g). Figure S36: Nitrogen sorption isotherms of [HoL(H_2_O)_2_]_n_·1.33DMF·2H_2_O (**7**). Filled symbols are for adsorption, empty symbols are for desorption (SBET = 286 m^2^/g). Figure S37: Fluorescence imaging of representative samples (Ligand; C1–7) excited with (a) green light, or (b) UV Objective 20x. Table S2: Bond distances (Å) and angles (°) for shI_3971_BeDa. Table S3: Bond distances (Å) and angles (°) for shI_3972_BeDa. Table S4: Bond distances (Å) and angles (°) for shI_3984_BeDa.
