# Supplementary material for: New Microporous Lanthanide Organic Frameworks. Synthesis, Structure, Luminescence, Sorption, and Catalytic Acylation of 2-Naphthol
Source: Molecules. 2020 Jul 3;25(13):3055. doi: 10.3390/molecules25133055 (PMC7411860; doi:10.3390/molecules25133055)
Supplement: Supplementary file 1 [file molecules-25-03055-s001.pdf]

Electronic supplementary material for

New microporous lanthanide organic frameworks. Synthesis, structure, luminescence, sorption, and catalytic acylaton of 2-naphthol

Dana Bejan[1*], Lucian Gabriel Bahrin[1], Sergiu Shova[1], Narcisa Marangoci[1], Ülkü Kökçam-Demir[2], Vasile Lozan[1,3*], Christoph Janiak[2*]

[a1] "Petru Poni" Institute of Macromolecular Chemistry, Gr. Ghica Voda Alley, no. 41A, RO 700487, Iasi, Romania

[2]Institut für Anorganische Chemie und Strukturchemie, Heinrich-Heine Universität Düsseldorf, Universitätsstr. 1, D 40225 Düsseldorf, Germany.

[3]Institute of Chemistry of MECR, Academiei str. 3, MD2028, Chisinau, Republic of Moldova

Dana Bejan	bejan.dana@icmpp.ro
Lucian Gabriel Bahrin	bahrin.lucian@icmpp.ro
Sergiu Shova	shova@icmpp.ro
Narcisa Laura Marangoci	nmarangoci@icmpp.ro
Ülkü Kökçam-Demir	Uelkue.Koekcam@uni-duesseldorf.de
Vasile Lozan	lozan.vasile@icmpp.ro
Janiak Cristoph	janiak@uni-duesseldorf.de

Content

1. Synthesis and microscopic images P2 – P4
2. Infrared spectroscopy(ATR) P4 – P8
3. Powder X-ray Diffraction P8 - P13
4. Thermal (TG/DTG) Analysis P13 - P16
5. Gas sorption izotherm P17 - P20
6. Fluorescence P21 – P22
7. Catalytic experiments P23
8. Crystallographic Data P23-P28

1. Synthesis of the ligand 1,3,5-tris(4-carboxyphenyl)-2,4,6-trimethylbenzene (H_3L)

In a 250 mL three-necked Schlenk flask, K_2CO_3 (6.55 g, 47 mmol) was dissolved in 30 mL of H_2O. To this solution, under vigourous stirring, 4-carboxyphenylboronic acid (1.94 g, 11.7 mmol) and triiodomesitylene (1.49 g, 3 mmol) were added, followed by tetrakis(triphenylphosphine)-palladium(0) (0.45 g, 0.38 mmol) in dioxane (50 mL) and EtOH (30 mL). This mixture was degassed by bubbling N_2 for 30 min and then refluxed under N_2 for 72 h. After the desired reaction time, the mixture was filtered and the filtrate was neutralized with a solution of hydrochloric acid (1 mol/L) until a fine white precipitate was formed. The product was isolated by centrifugation, washed several times with water and recrystallized (Yield 50 %, colorless crystals).

***Synthesis of compound [LaL(H₂O)₂]ₙ* (1)**

$La(NO_3)_3{\cdot}6H_2O$ (0.036 g, 0.08 mmol) and H_3L (0.01 g, 0.02 mmol) were dissolved in ethanol and water (2.5 mL/0.5 mL) at room temperature. The solution was transferred into a 20 mL culture tube and kept under static conditions for 5 days at 80 °C. After cooling, the colorless crystalline product was collected by centrifugation and washed with ethanol. Finally, the crystals were dried at room temperature (0.008 g).

***Synthesis of compound $[CeL(H_2O)_2]_n$* (2)**

2 was prepared following the synthetic procedure for 1 using $Ce(NO_3)_3{\cdot}6H_2O$ (0.039 g, 0.08 mmol), H_3L (0.01 g, 0.02 mmol), deionized water (0.5 mL) and ethanol (2.5 mL). The colorless crystals were dried at room temperature (0.012 g).

Synthesis of compound $[NdL(H_2O)_2]{\cdot}1.33DMF{\cdot}2H_2O$ (3)

To a solution of H_3L (0.01 g, 0.02 mmol) in DMF (5.0 mL), in a 20 mL culture tube was added $Nd(NO_3)_3{\cdot}6H_2O$ (0.036 g, 0.08 mmol) dissolved in ethanol/water (2.5 mL/0.5 mL) at room temperature. The tube with clear solution was kept under static conditions for 1 day at 80 °C. After cooling, the violet crystalline product was collected by centrifugation and washed with DMF and ethanol. Finally, the crystals were dried at room temperature (0.0138g).

Synthesis of compound $[EuL(H_2O)_2]{\cdot}1.33DMF{\cdot}2H_2O$ (4)

$Eu(NO_3)_3{\cdot}6H_2O$ (0.04 g, 0.08 mmol) and H_3L (0.01 g, 0.02 mmol) were dissolved in DMF and ethanol (0.5 mL/2.5 mL) at room temperature. The solution was kept under static conditions for 4 days at 80 °C. After cooling, the colorless crystalline product was collected by centrifugation and washed with DMF and then with ethanol. Finally, the crystals were dried at room temperature (0.01 g).

***Synthesis of compound $[GdL(H_2O)_2]{\cdot}2DMF{\cdot}2H_2O$* (5)**

A solution containing H_3L (0.0298 g, 0.06 mmol), $Gd(NO_3)_3{\cdot}6H_2O$ (0.085 g, 0.24 mmol), DMF (1.5 mL), ethanol (4 mL) and water (1.5 mL) was transferred into a tube and kept under static conditions for 2 day at 80 °C. The white crystalline product was isolated and dried at room temperature (0.04 g).

***Synthesis of compound $[DyL(H_2O)_2]{\cdot}$*(6)**

6 was prepared following the synthetic procedure for 3 using: $Dy(NO_3)_3{\cdot}6H_2O$ (0.093 g, 0.26 mmol), H_3L (0.032 g, 0.06 mmol) in DMF/ethanol/water (1.5 mL/4 mL/0.75 mL). The tube

with clear solution was kept under static conditions for 3 day at 80 °C. The white crystals were dried at room temperature (0.03 g).

***Synthesis of compound [HoL(H_2O)$_2$]·1.33DMF·2H_2O* (7)**

7 was prepared following the synthetic procedure for **3** using: Ho(NO_3)$_3$·5H_2O (0.09 g, 0.2 mmol), H_3L (0.032 g, 0.06 mmol) in DMF/ethanol/water (1.5 mL/4 mL/0.75 mL). The crystalline product was isolated and dried at room temperature (0.02 g).

Table S1. Overview of the experimental details for the synthesis of **1-7**.

**Compound**	**DMF (mL)**	**EtOH (mL)**	**H_2O (mL)**	**Time (d)**	**T (°C)**	**Yield (%)**
1	-	2.5	0.5	5	80	62
2	-	2.5	0.5	5	80	88
3	5	2.5	0.5	1	80	84
4	0.5	2.5	-	4	80	66
5	1.5	4	1.5	2	80	82
6	1.5	4	0.75	3	80	77
7	1.5	4	0.75	3	80	45

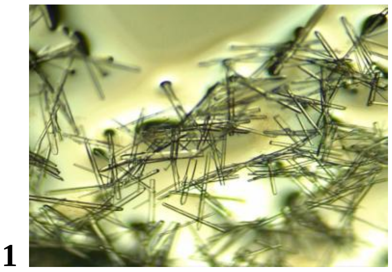

1

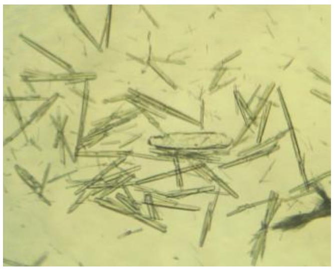

2

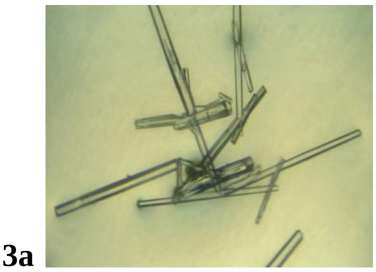

3a

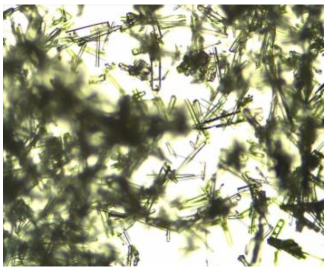

3b

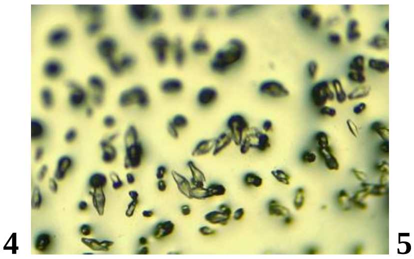

4

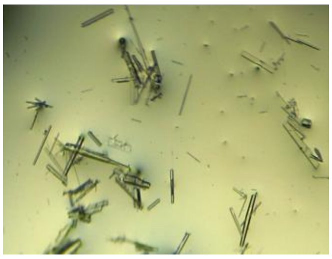

5

6 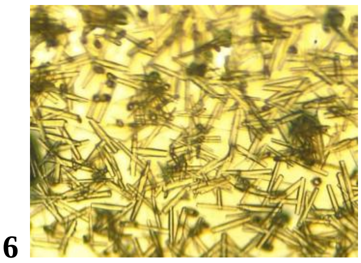 7 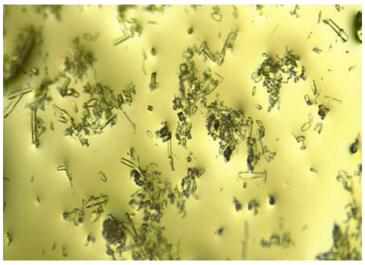

Figure S1: Optical microscopy image of **1-7**, with Leica ICC50 W, 4x/0.10.

2. Infrared spectroscopy (ATR)

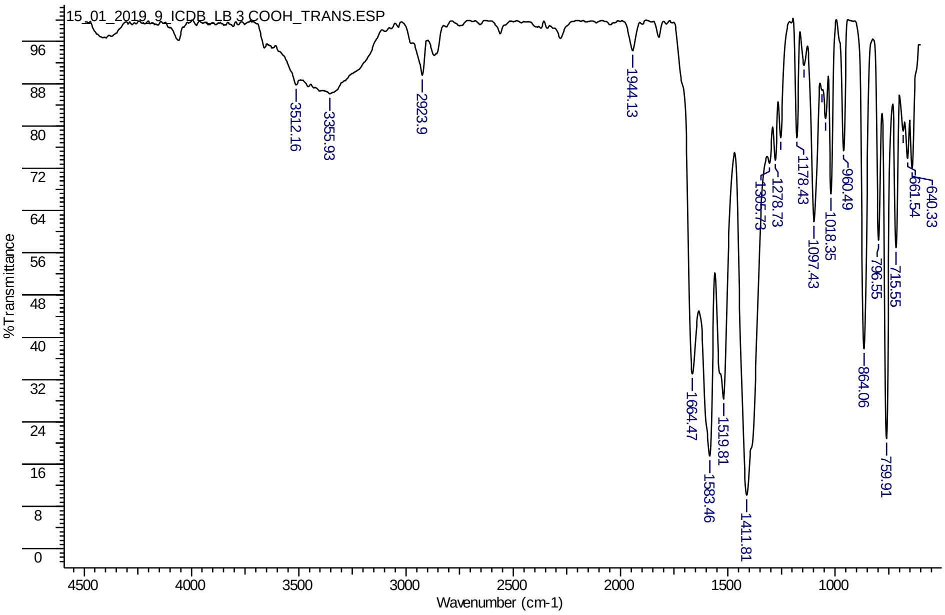

Figure S2. IR spectrum of ligand **H_3L.**

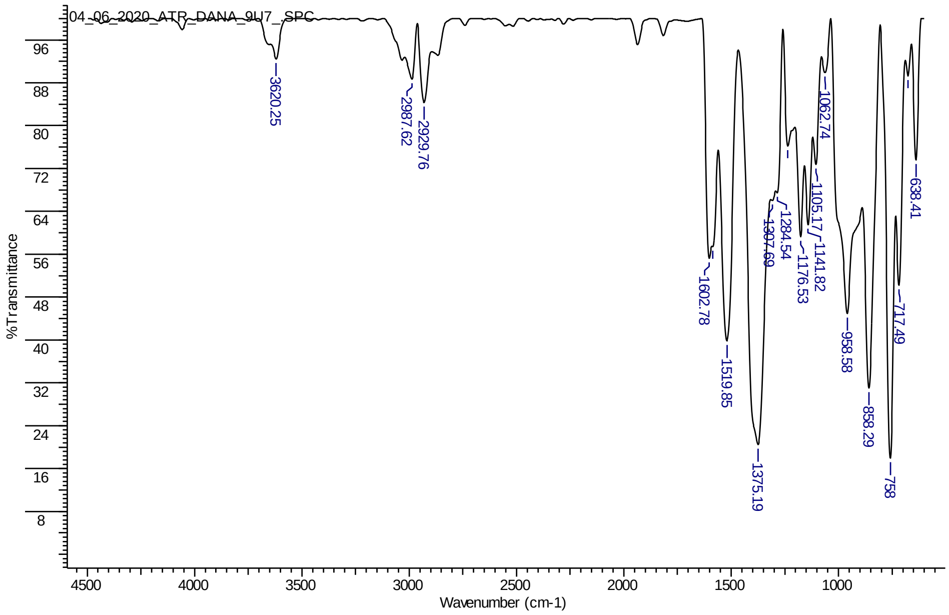

Figure S3. IR spectrum of compound $[LaL(H_2O)_2]_n$ **(1)**.

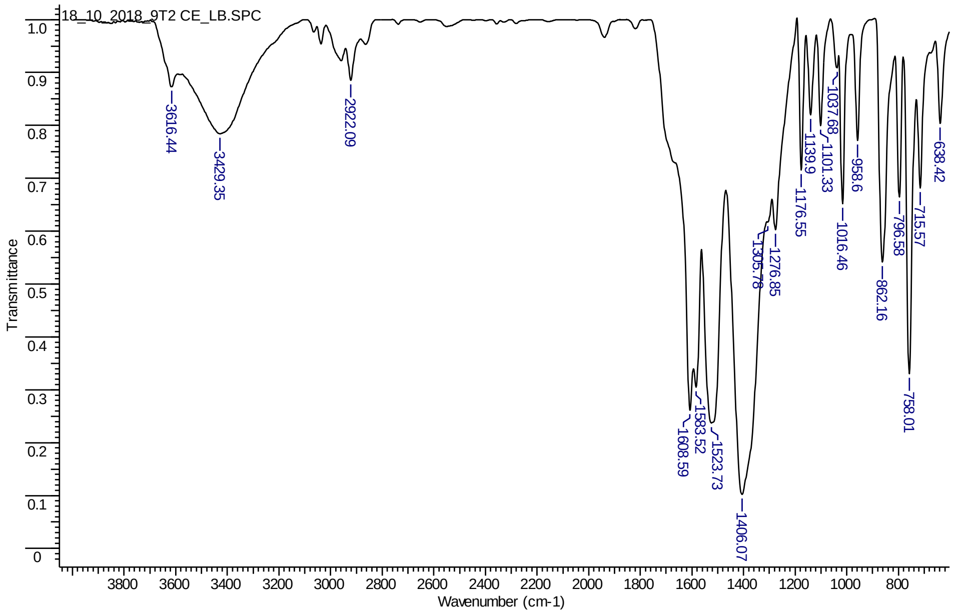

Figure S4. IR spectrum of compound $[CeL(H_2O)_2]_n$ **(2)**.

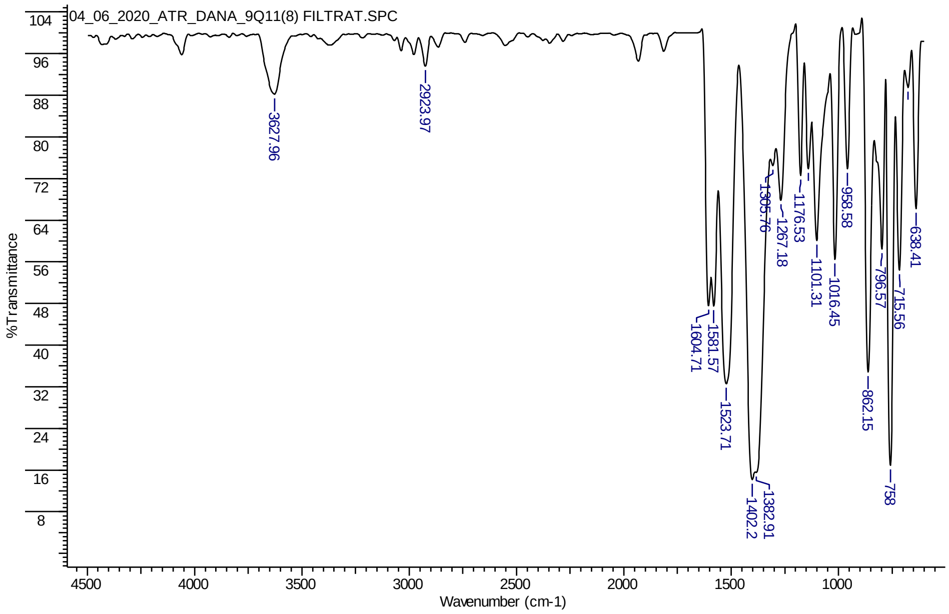

Figure S5. IR spectrum of compound $[NdL(H_2O)_2]_n\cdot 1.33DMF\cdot H_2O$ (**3**).

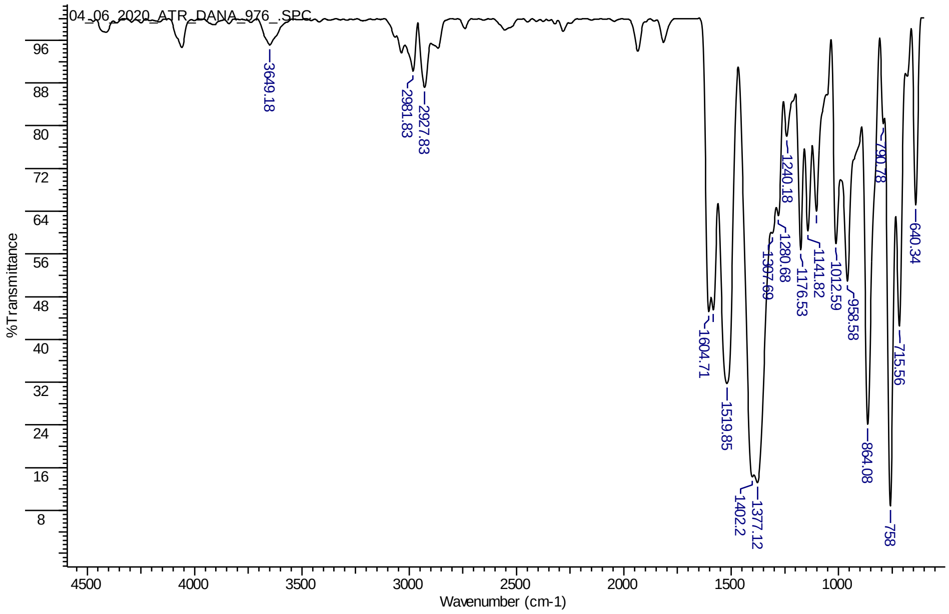

Figure S6. IR spectrum of compound $[EuL(H_2O)_2]_n\cdot 1.33DMF\cdot 2H_2O$ (**4**).

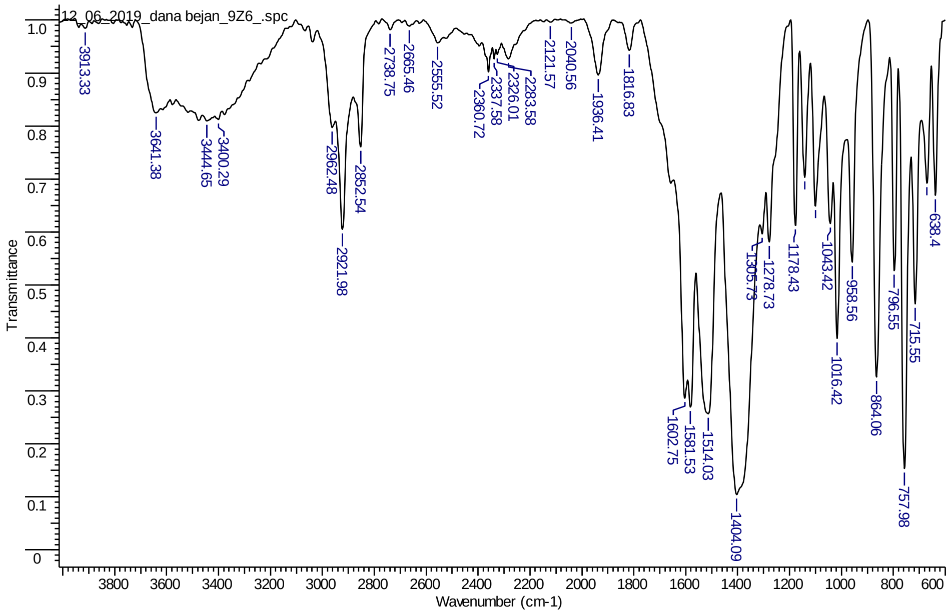

Figure S7. IR spectrum of compound [GdL(H_2O)$_2$]$_n$ ·2DMF 2H_2O(**5**).

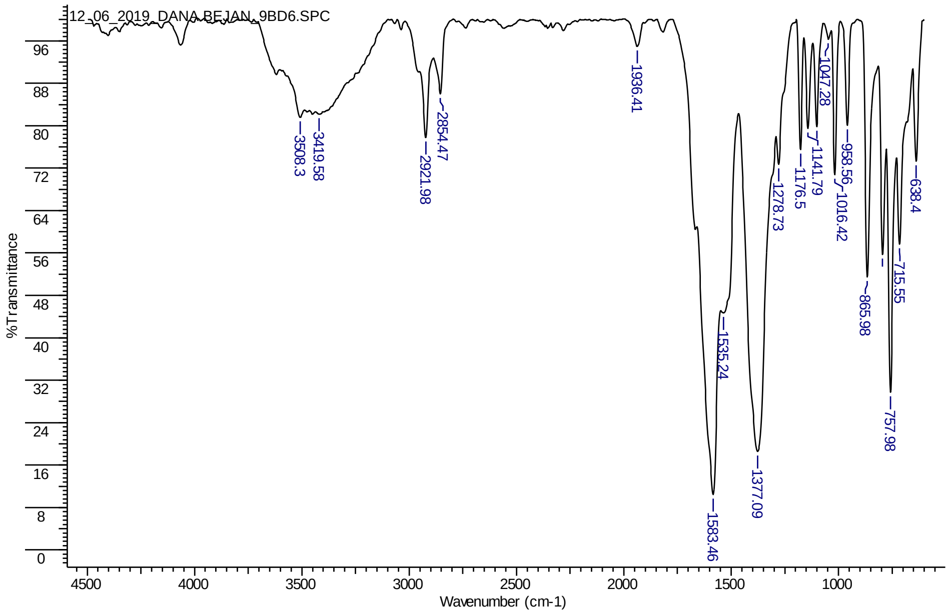

Figure S8. IR spectrum of compound [DyL(H_2O)$_2$]$_n$ (**6**).

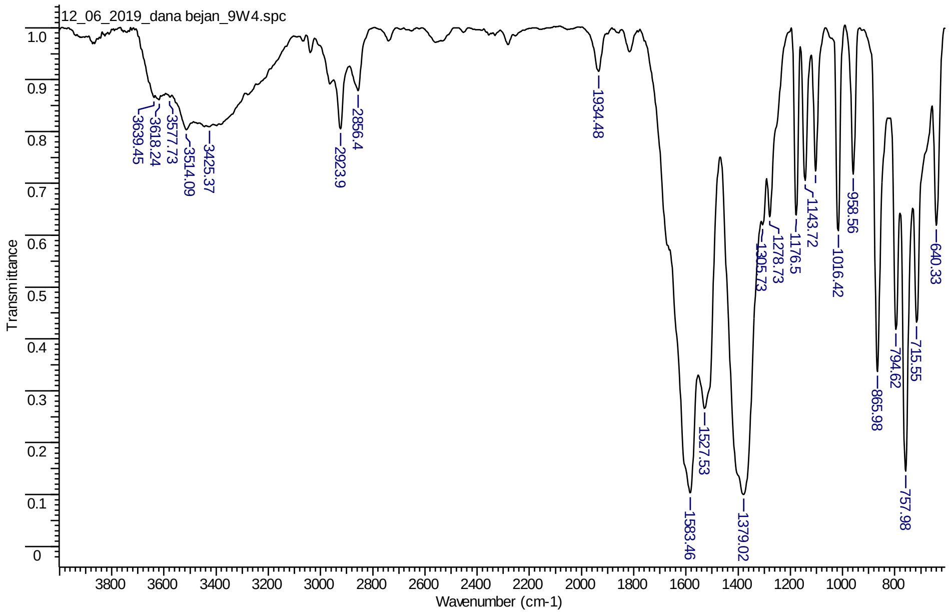

Figure S9. IR spectrum of compound $[HoL(H_2O)_2]_n \cdot 1.33DMF \cdot 2H_2O$ (**4**).

3. Powder X-ray Diffraction (PXRD)

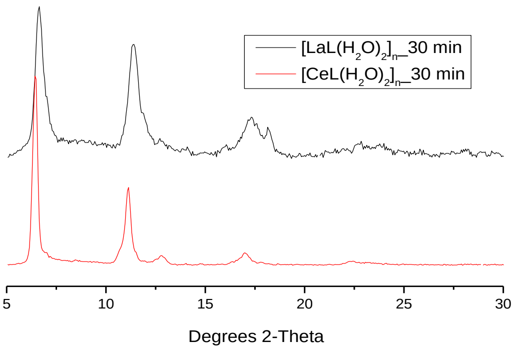

Figure S10. PXRD patterns of the two isostructural compounds $[LaL(H_2O)_2]_n$ (**1**) and $[CeL(H_2O)_2]_n$ (**2**) recorded at different time (6 min and 30 min).

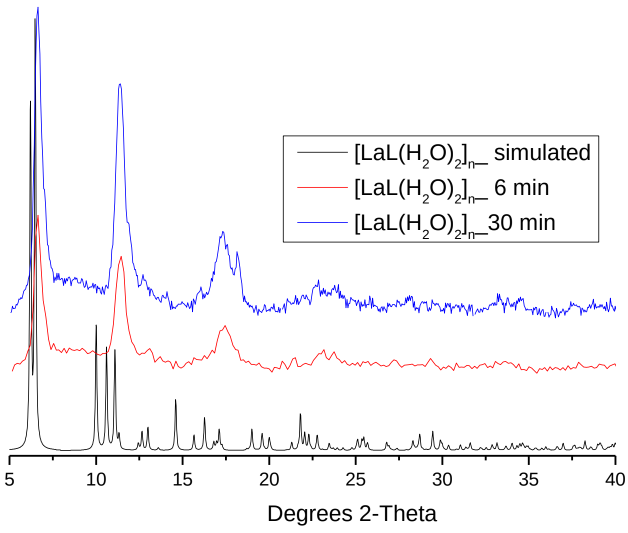

Figure S11. PXRD patterns of $[LaL(H_2O)_2]_n$ (**1**) recorded at different time. (6 min and 30 min).

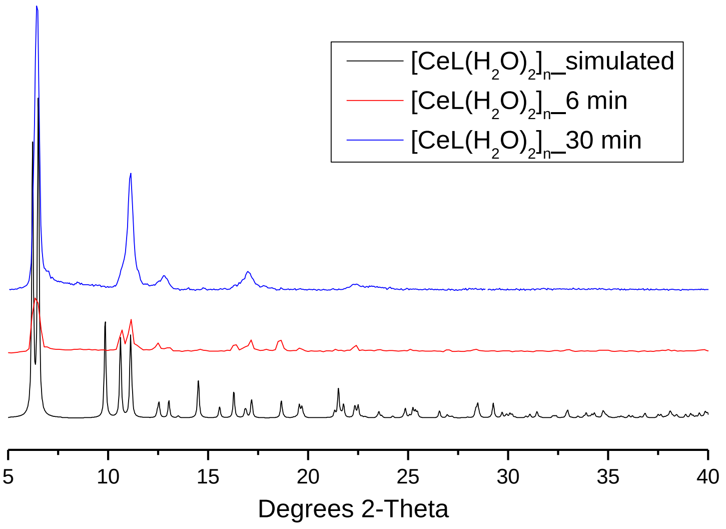

Figure S12. PXRD patterns of $[CeL(H_2O)_2]_n$ (**2**).

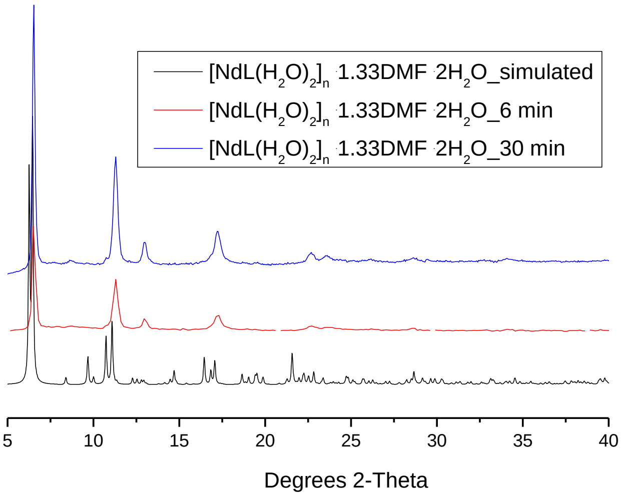

Figure S13. PXRD patterns of $[NdL(H_2O)_2]_n{\cdot}1.33DMF{\cdot}H_2O$ (**3**).

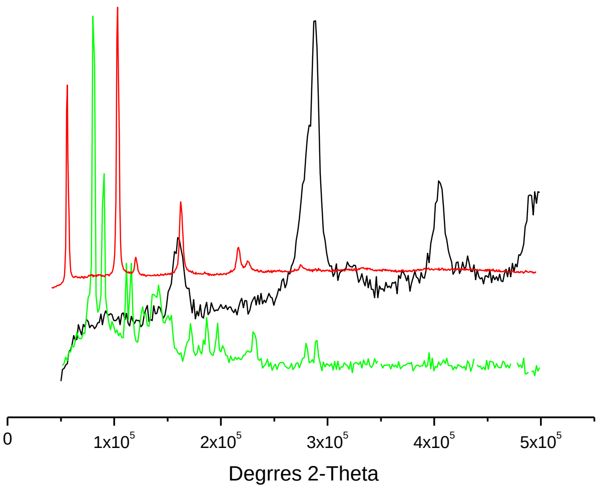

Figure S14. PXRD patterns of compound $[NdL(H_2O)_2]_n \cdot 1.33DMF \cdot H_2O$ (**3**)-red, compound **3** after basic condition- black; compound **3** after acidic condition- green.

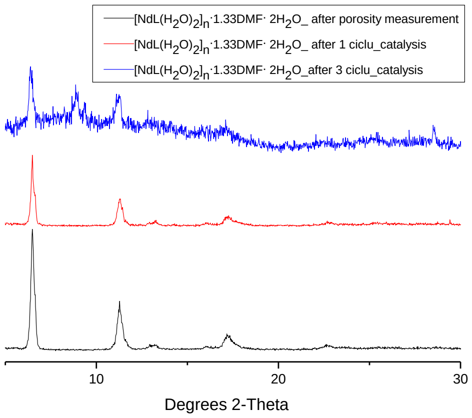

Figure S15. PXRD patterns of activated compound $[NdL(H_2O)_2]_n \cdot 1.33DMF \cdot H_2O$ (**3**) - black and compound (**3**) after catalytic reaction (first cycle) – red and (**3**) – blue, after third cycle in catalytic processes.

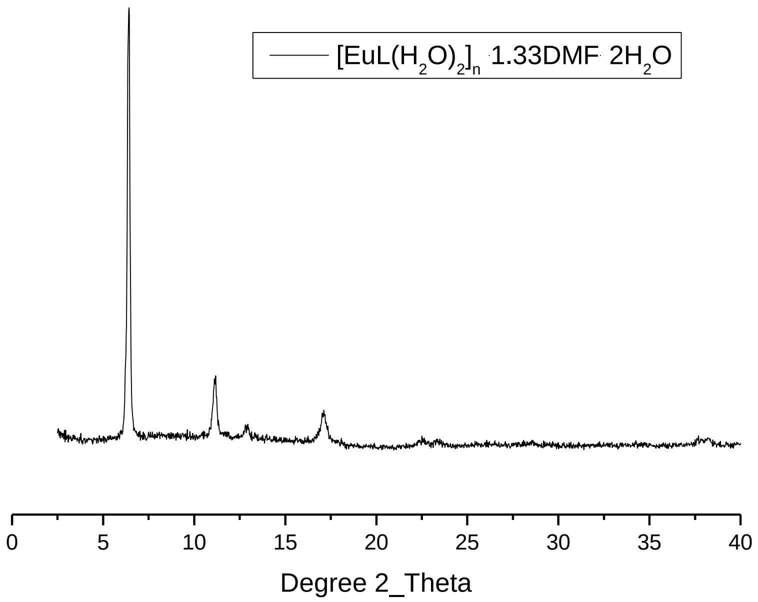

Figure S16. PXRD patterns of $[EuL(H_2O)_2]_n \cdot 1.33DMF \cdot 2H_2O$ (**4**).

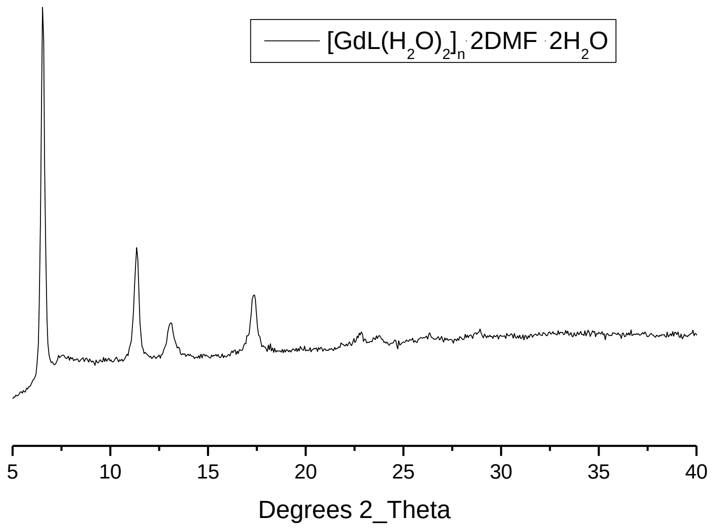

Figure S17. PXRD patterns of $[GdL(H_2O)_2]_n \cdot 2DMF\ 2H_2O$(**5**).

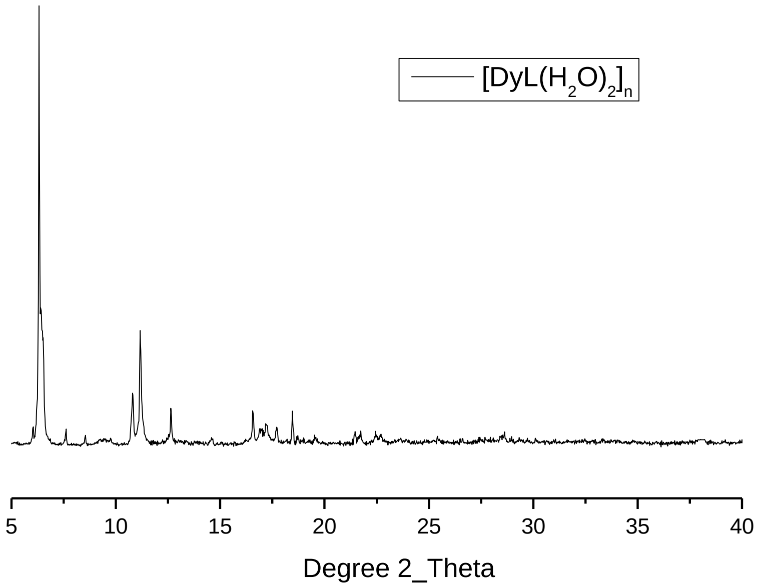

Figure S18. PXRD patterns of [DyL(H_2O)$_2$]$_n$ (**6**).

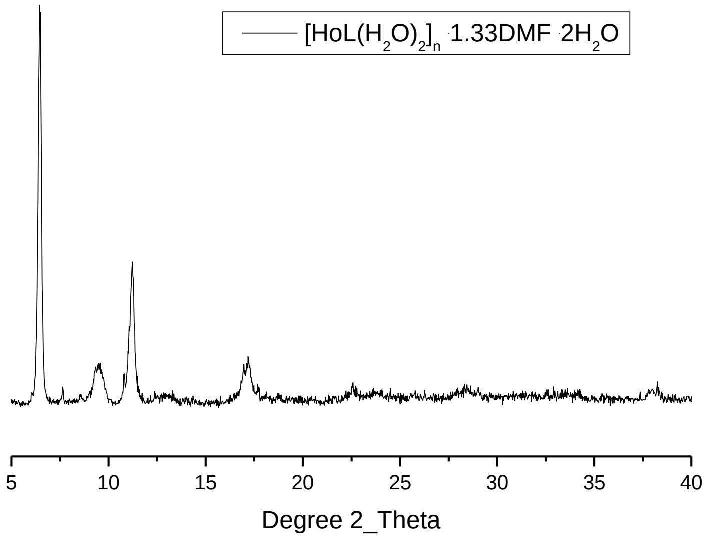

Figure S19. PXRD patterns of [HoL(H_2O)$_2$]$_n$·1.33DMF·H_2O (**7**).

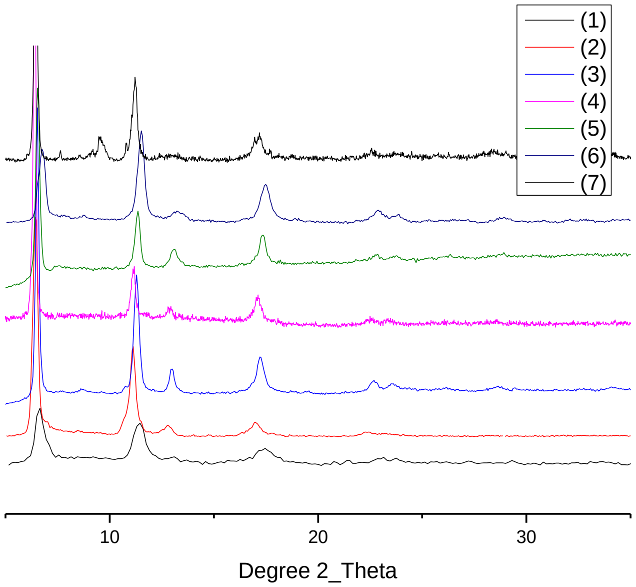

Figure S20. PXRD patterns of all compounds (**1-7**).

4. Thermal (TG/DTG) Analysis

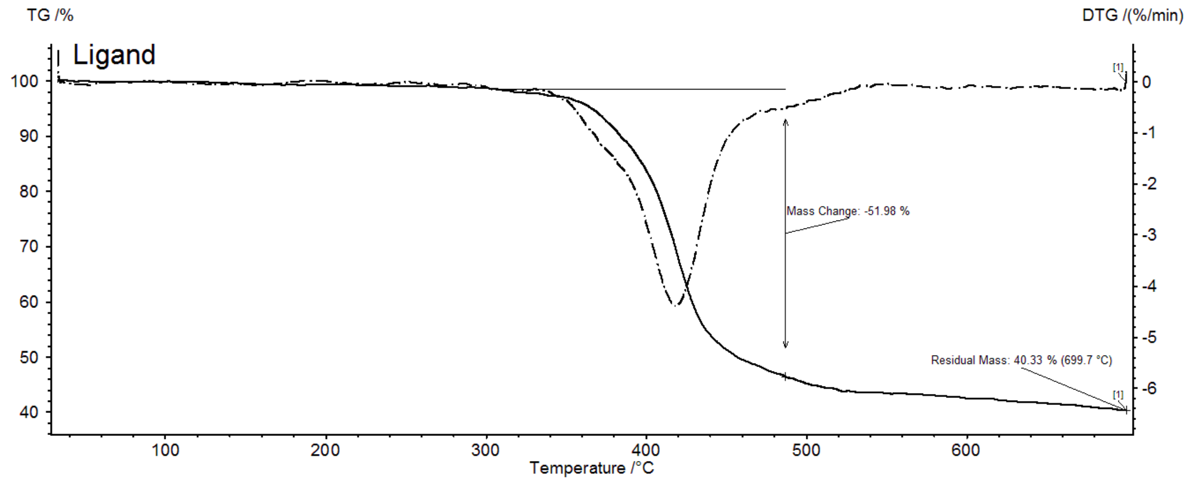

Figura S21. Thermogravimetric analysis of ligand **H_3L.**

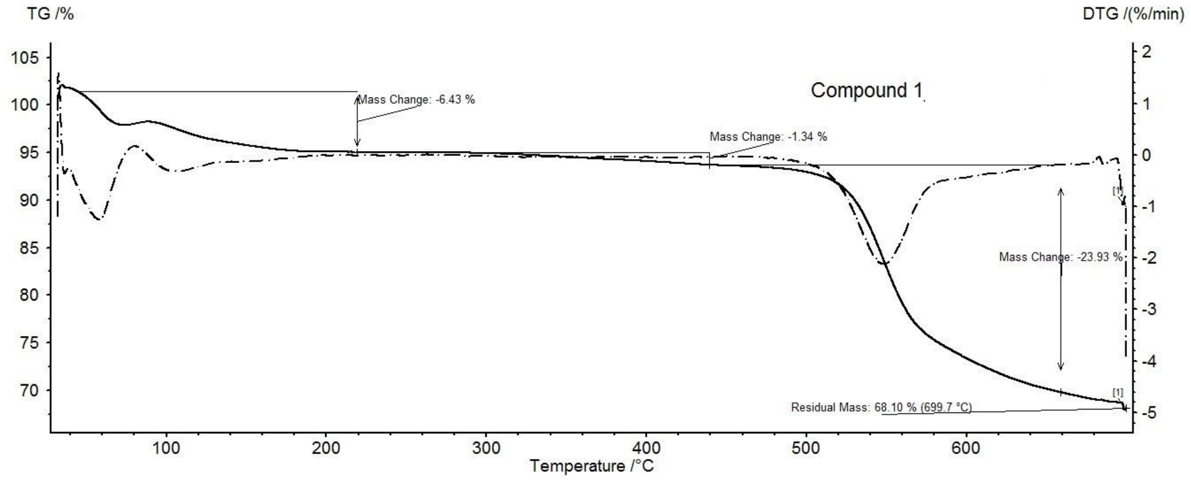

Figura S22. Thermogravimetric analysis of compound $[LaL(H_2O)_2]_n$ (**1**).

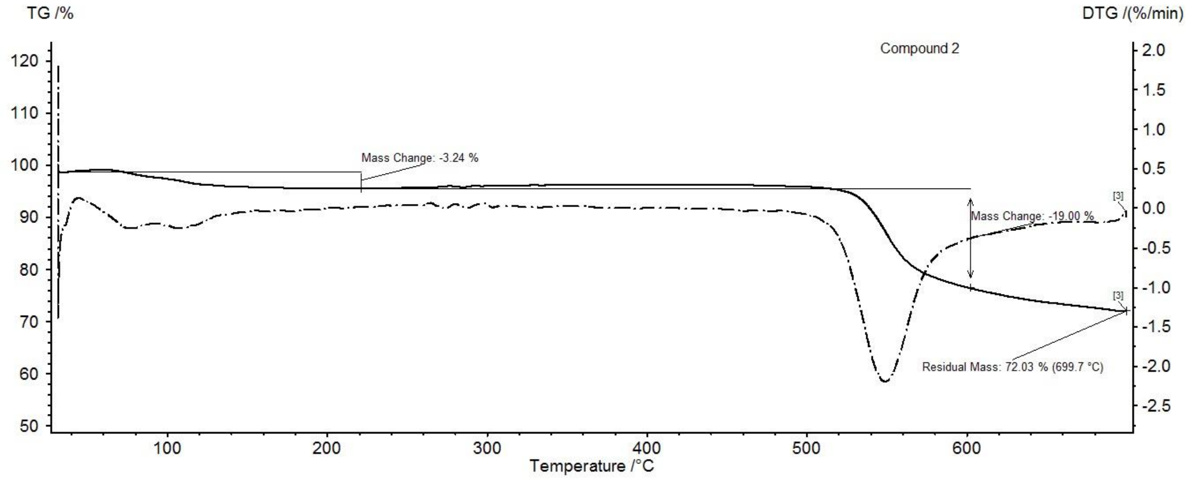

Figura S23. Thermogravimetric analysis of compound $[CeL(H_2O)_2]_n$ (**2**).

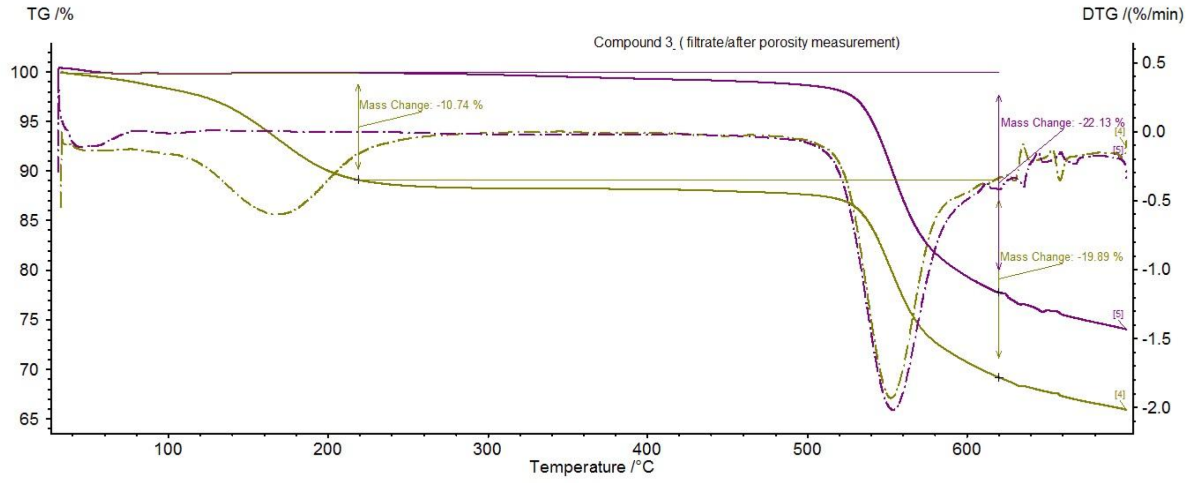

Figura S24. Thermogravimetric analysis of compound $[NdL(H_2O)_2]_n \cdot 1.33DMF \cdot H_2O$ (**3**).

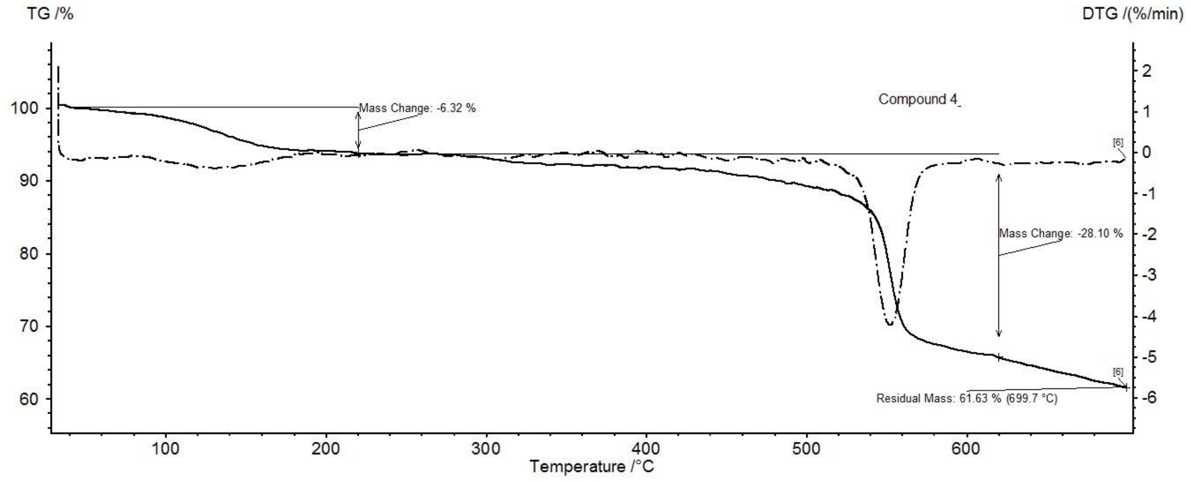

Figura S25. Thermogravimetric analysis of compound $[EuL(H_2O)_2]_n \cdot 1.33DMF \cdot 2H_2O$ (**4**).

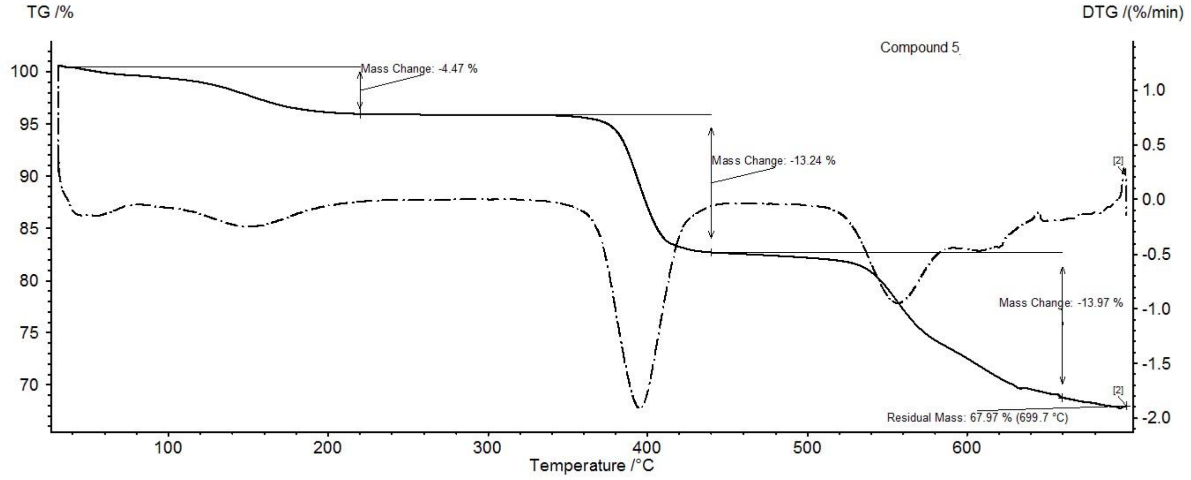

Figure S26. Thermogravimetric analysis of compound $[GdL(H_2O)_2]_n \cdot 2DMF\ 2H_2O$(**5**).

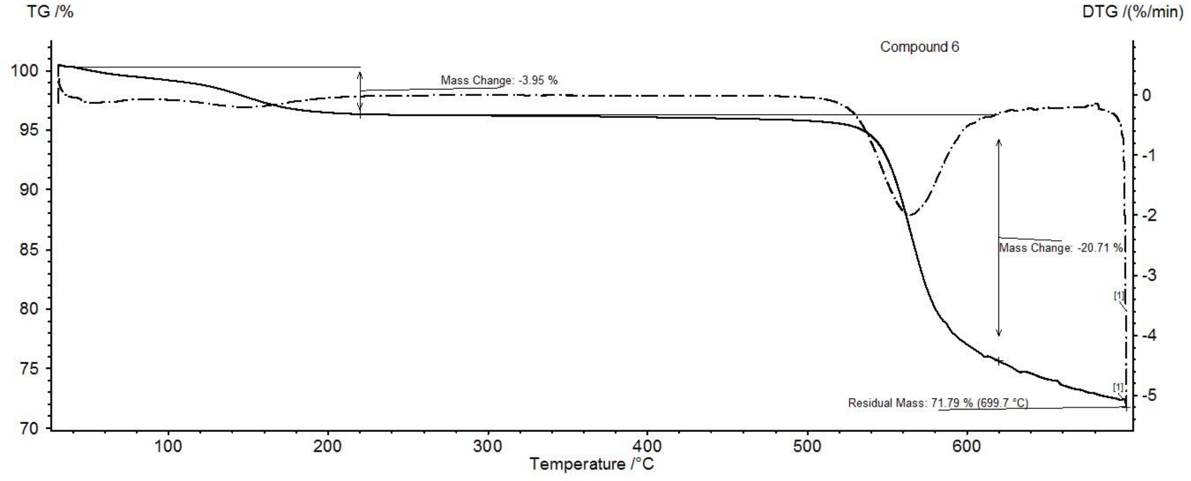

Figure S27. Thermogravimetric analysis of compound $[DyL(H_2O)_2]_n$ (**6**).

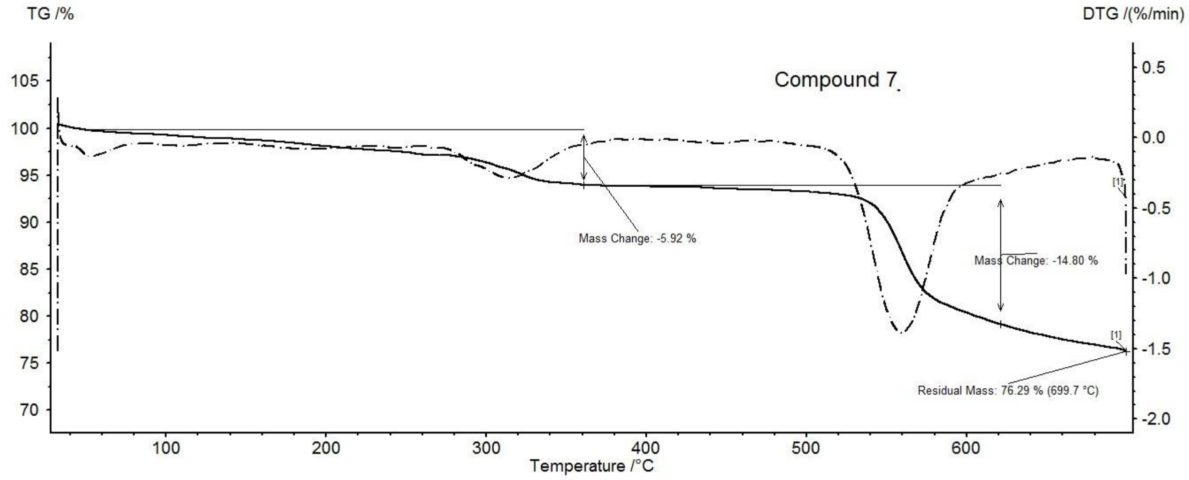

Figure S28 Thermogravimetric analysis of compound $[HoL(H_2O)_2]_n·1.33DMF·H_2O$ (**7**).

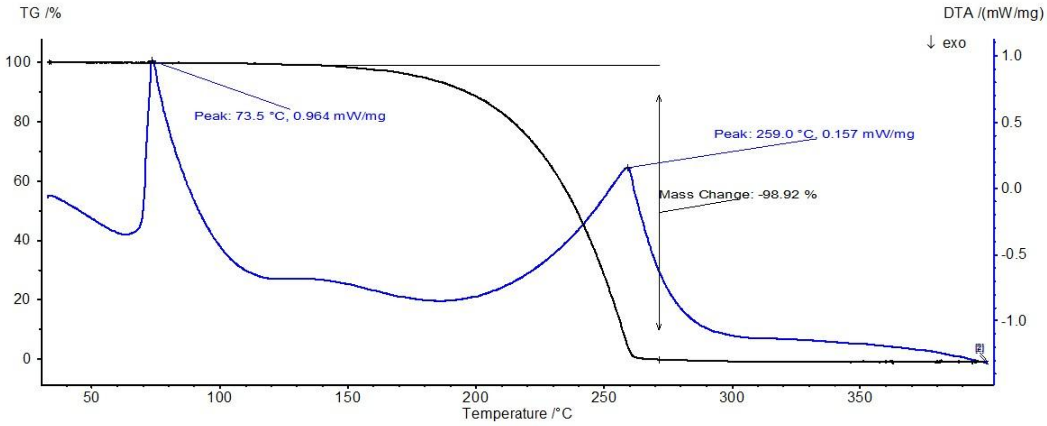

Figure S29. Thermogravimetric analysis of 2-naphyl acetate.

5. Gas sorption isotherms

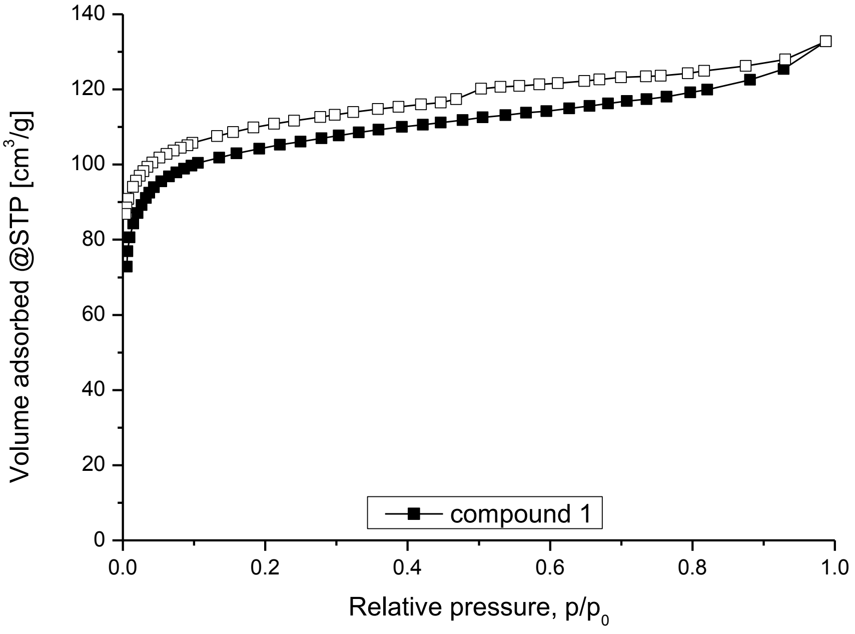

Figure S30. Nitrogen sorption isotherms of $[LaL(H_2O)_2]_n$ (**1**) recorded at 77 K. Filled symbols are for adsorption, empty symbols are for desorption (S_{BET} = 405 m^2/g).

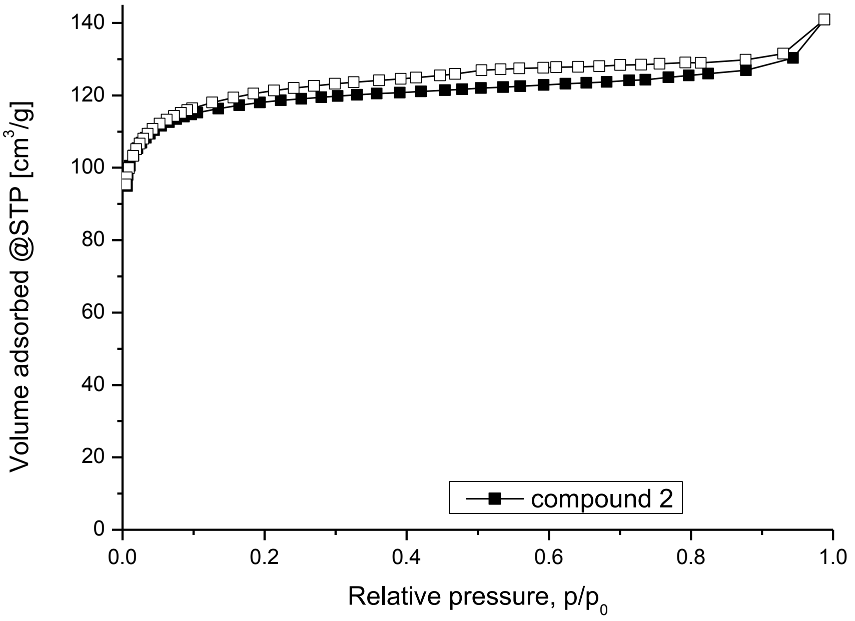

Figure S31. Nitrogen sorption isotherms of $[CeL(H_2O)_2]_n$ (**2**) recorded at 77 K. Filled symbols are for adsorption, empty symbols are for desorption (S_{BET} = 467 m^2/g).

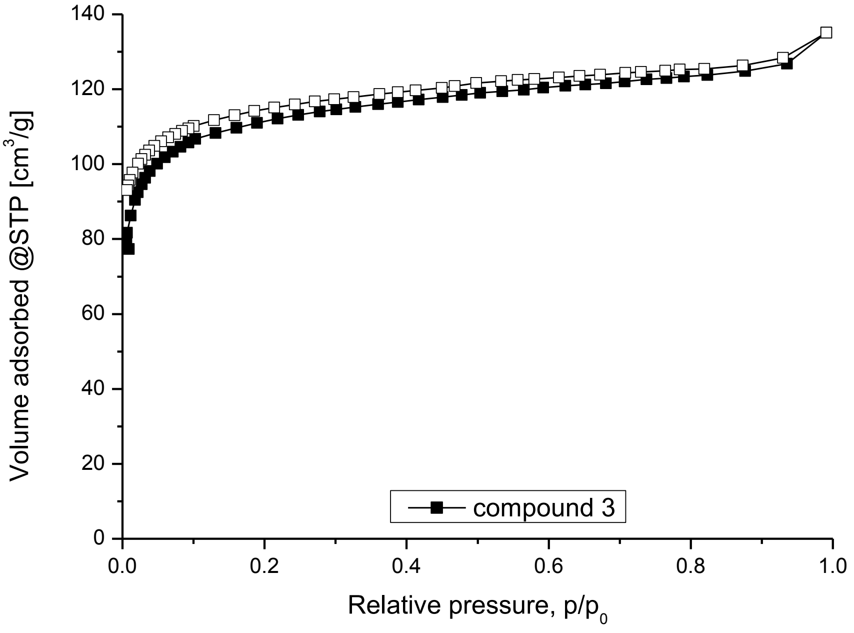

Figure S32. Nitrogen sorption isotherms of $[NdL(H_2O)_2]_n \cdot 1.33DMF \cdot H_2O$ (**3**) recorded at 77 K. Filled symbols are for adsorption, empty symbols are for desorption (S_{BET} = 426 m^2/g).

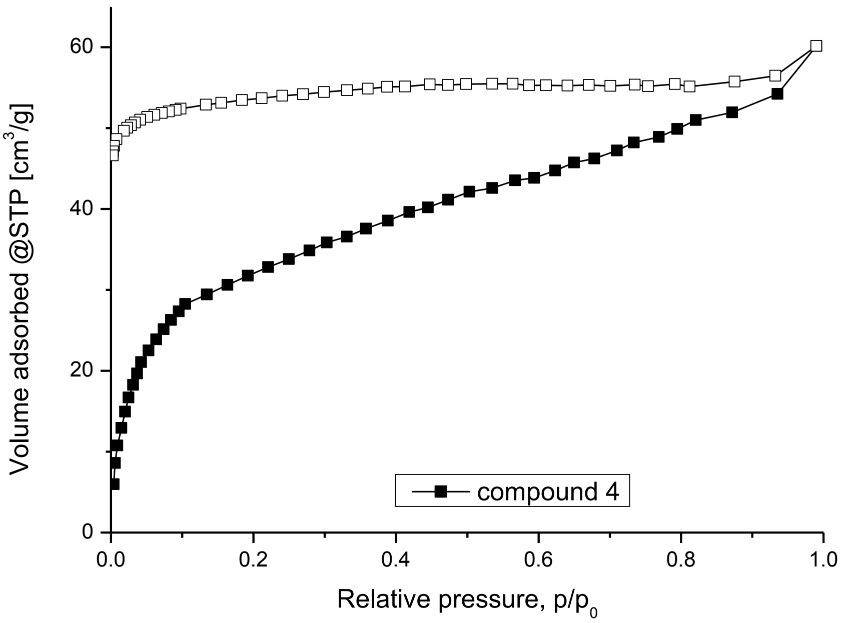

Figure S33. Nitrogen sorption isotherms of $[EuL(H_2O)_2]_n \cdot 1.33DMF \cdot 2H_2O$ (**4**) recorded at 77 K. Filled symbols are for adsorption, empty symbols are for desorption (S_{BET} = 114 m^2/g).

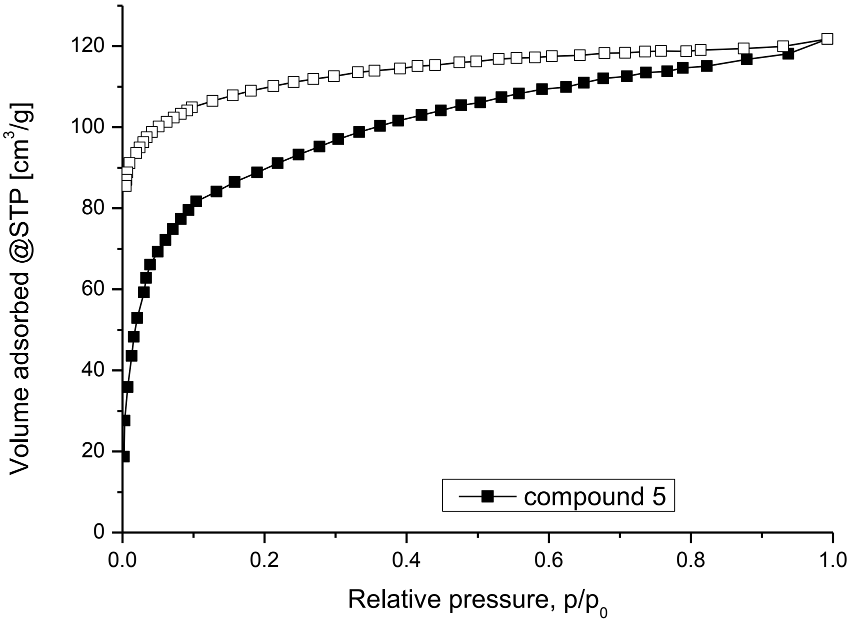

Figure S34 . Nitrogen sorption isotherms of $[GdL(H_2O)_2]_n \cdot 2DMF\ 2H_2O$(**5**) recorded at 77 K. Filled symbols are for adsorption, empty symbols are for desorption (S_{BET} = 348 m^2/g).

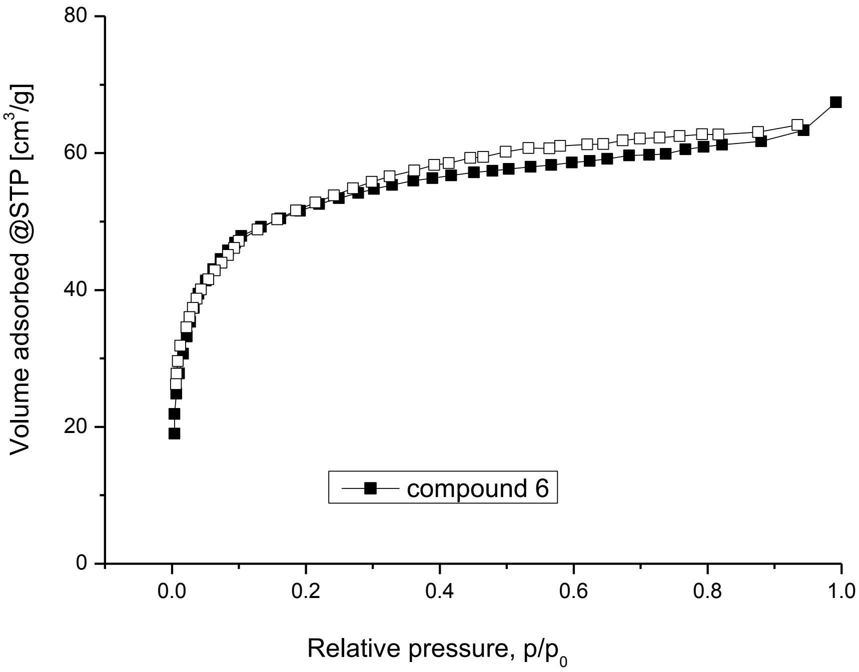

Figure S35a. Nitrogen sorption isotherms of $[DyL(H_2O)_2]_n$ (**6**) recorded at 77 K. Filled symbols are for adsorption, empty symbols are for desorption (S_{BET} = 202 m^2/g).

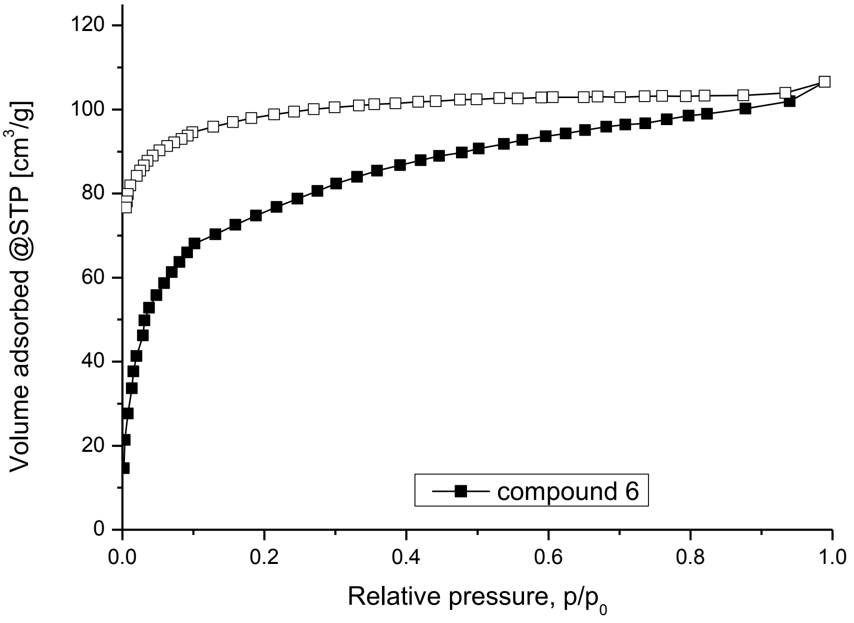

Figure S35b. Nitrogen sorption isotherms of $[DyL(H_2O)_2]_n$ (**6**) recorded at 77 K. (different synthesis, the same condition). Filled symbols are for adsorption, empty symbols are for desorption (S_{BET} = 298 m^2/g).

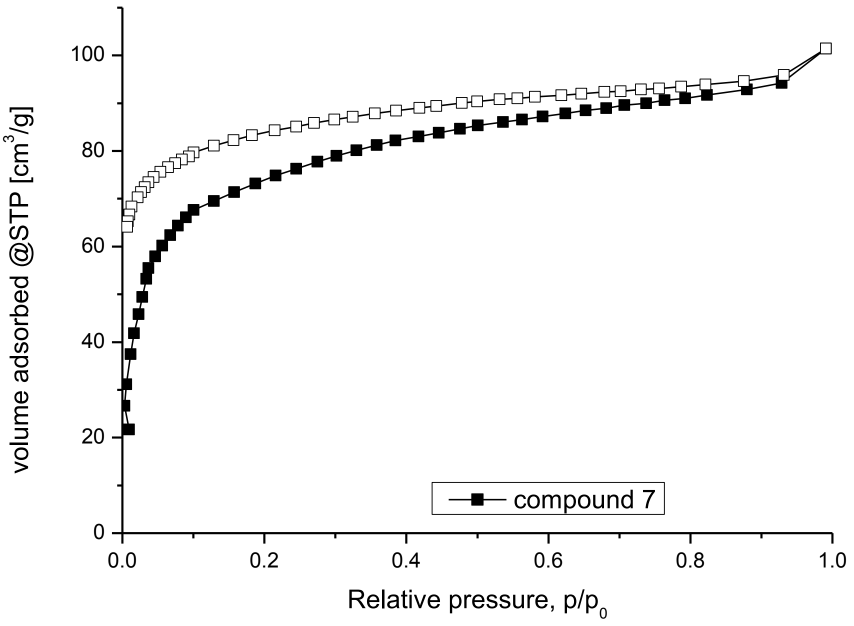

Figure S36. Nitrogen sorption isotherms of $[HoL(H_2O)_2]_n$·1.33DMF·H_2O (**7**) .Filled symbols are for adsorption, empty symbols are for desorption (S_{BET} = 286 m^2/g).

6. Fluorescence

Ligand
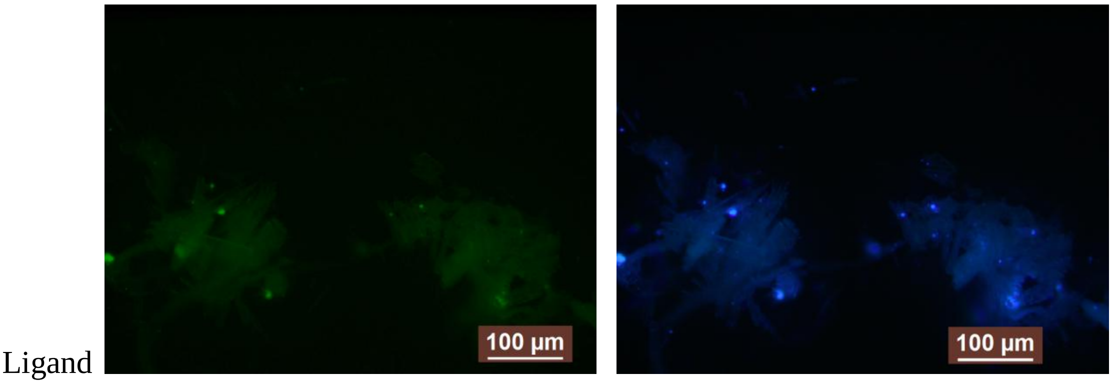

(b)

1 (a)
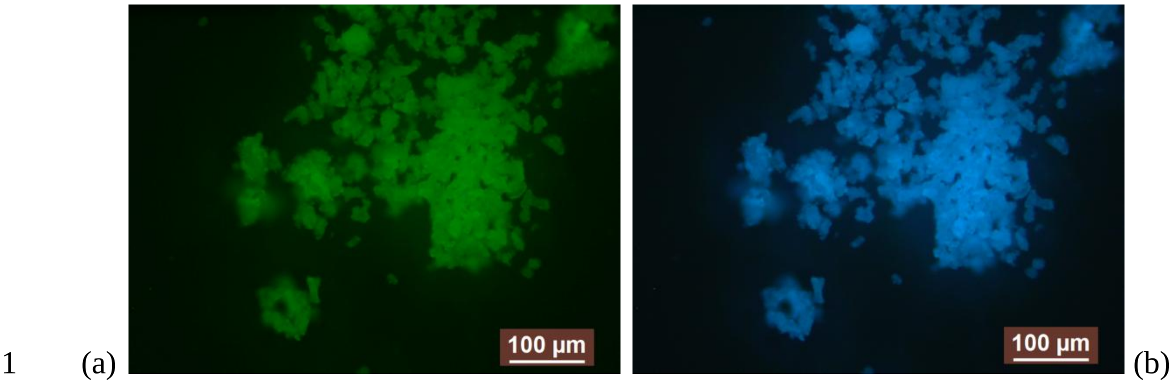

(b)

2 (a)
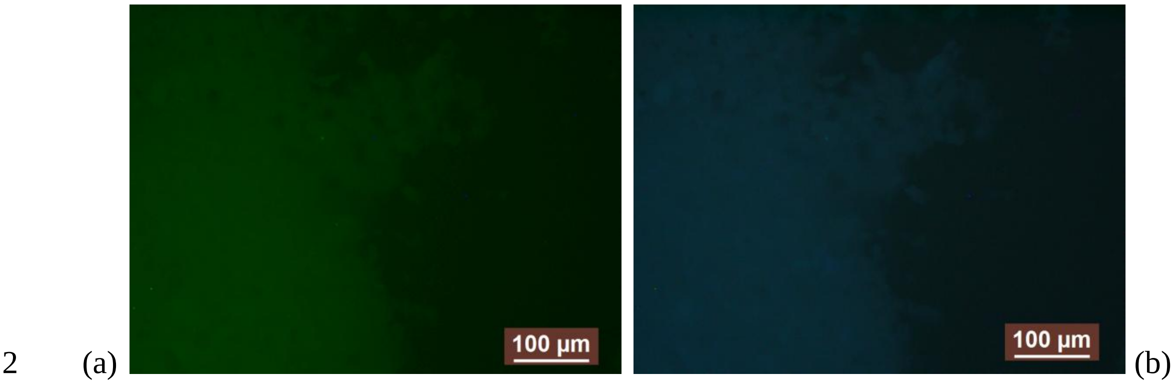

(b)

3 (a)
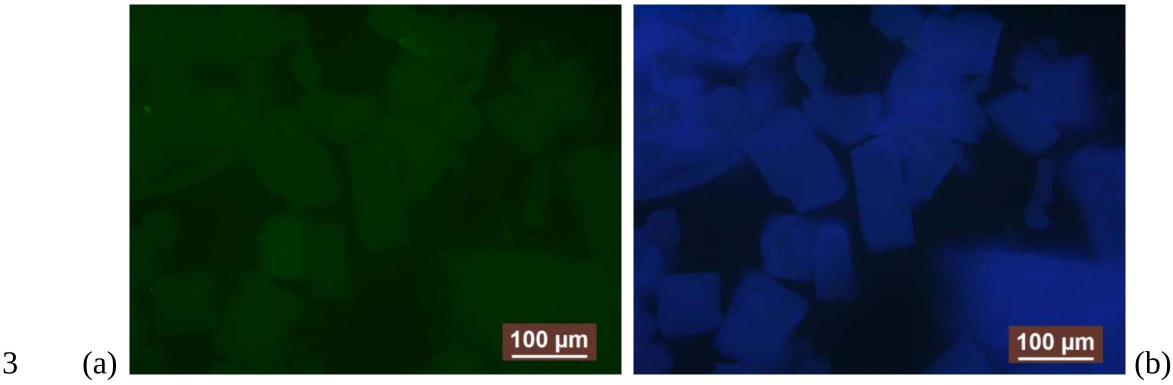

(b)

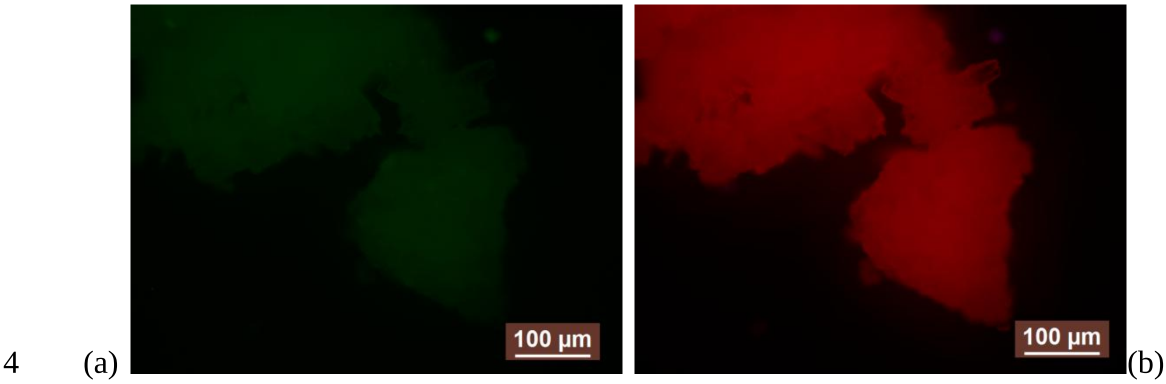

4 (a) (b)

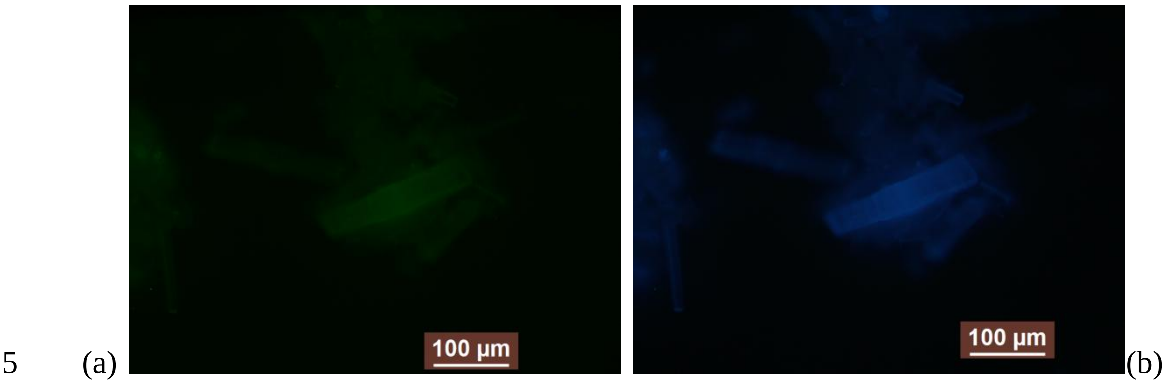

5 (a) (b)

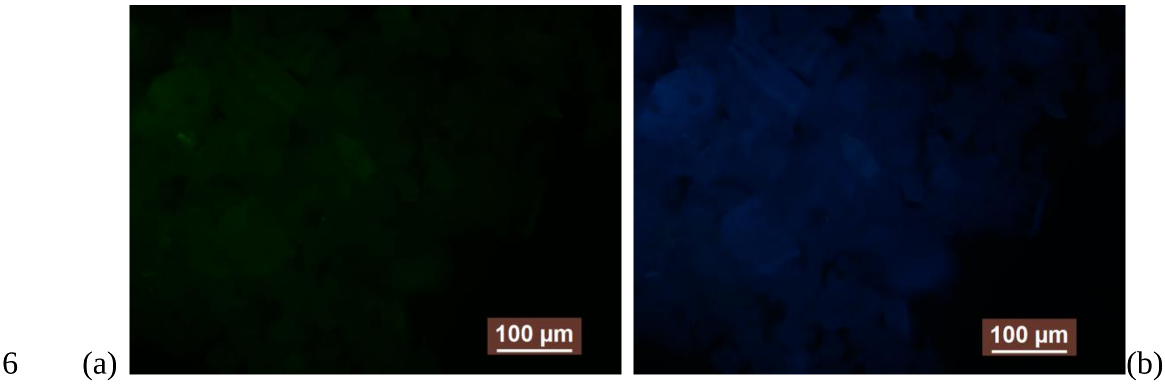

6 (a) (b)

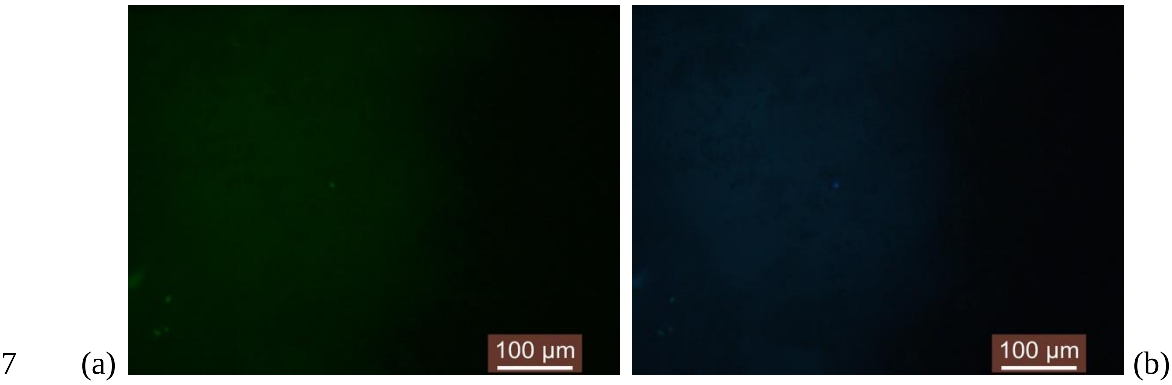

7 (a) (b)

Figure S37. Fluorescence imaging of representative samples (Ligand; C1-7): a) green light (470 nm), or b). UV (365 nm), Objective 20x.

7. Catalytic experiments

The chemical reagents used in catalytic reaction were as follows: 2-naphthol (1 mmol), acetic anhydride (1.5 equiv.), catalyst (1 mmol%) and $CHCl_3$ (2 mL). The catalytic reaction was performed at room temperature for 24 hours. The catalyst was activated by exchange of the solvent with $CHCl_3$ (3·3 mL) at RT for 24 hours, followed by pore evacuation drying at 80 °C during of 20 hours.

Filtration test: After running the reaction for 5 hours, the catalyst material (**3**) was separated by centrifugation. The filtrate was separated and isolated (yield 65%). Then the reaction was repeated and after 5 hours the clear supernatant solution was filtered and the reaction was continued at room temperature for another 19 hours to a total run time of 24 h. The filtrate was isolated and analyzed and the yield of product had not increased to the yield of the separation after 5 h (both yields were 65%).

8. Crystallographic Data

Table S2. Bond distances (Å) and angles(°) for shI_3971_BeDa.

Ce1-O1^{1}	2.461(4)	C3-C4	1.385(6)
Ce1-O1*w*	2.524(3)	C5-C6	1.371(7)
Ce1-O2	2.467(4)	C6-C7	1.415(7)
Ce1-O3^{4}	2.574(3)	C8-C10	1.391(7)
O1-C1	1.256(6)	C10-C13	1.506(7)
O2-C1	1.252(6)	C11-C14	1.524(10)
O3-C18	1.270(4)	C14-C15	1.361(6)
C1-C2	1.493(7)	C15-C16	1.395(7)
C2-C3	1.383(7)	C16-C17	1.383(6)
C2-C7	1.371(7)	C17-C18	1.469(9)

$^{1)}$ – 1– *x*, – *y*, – 1 – *z*; $^{2)}$ ½ + *x*, + *y*, – 1– *z*; $^{3)}$ – ½ – *x*, – *y*, + *z*; $^{4)}$ – 1 – *x*, ½ + *y*, – ½ + *z*; $^{5)}$ ½ + *x*, – ½ – *y*, – ½ + z

C1-O1-Ce1^{1}	165.0(3)	C10-C8-C9	120.7(5)
C1-O2-Ce1	113.9(4)	C8-C9-C8^{3}	119.2(7)
C18-O3-Ce1^{2}	95.2(4)	C8-C9-C12	120.4(3)
O1-C1-C2	120.0(5)	C8-C10-C11	118.6(6)
O2-C1-O1	123.2(5)	C8-C10-C13	120.6(5)

O2-C1-C2	116.7(5)	C11-C10-C13	120.7(6)
C3-C2-C1	120.7(5)	C10-C11-C10^{3}	122.3(7)
C7-C2-C1	120.4(5)	C10-C11-C14	118.9(4)
C7-C2-C3	118.9(5)	C15-C14-C11	121.3(4)
C4-C3-C2	121.1(5)	C15^{3}-C14-C15	117.4(7)
C3-C4-C5	120.9(5)	C14-C15-C16	122.6(6)
C4-C5-C8	119.7(5)	C17-C16-C15	119.0(6)
C6-C5-C4	117.5(5)	C16-C17-C16^{3}	119.4(7)
C6-C5-C8	122.7(5)	C16-C17-C18	120.3(4)
C5-C6-C7	121.7(5)	O3-C18-Ce1^{2}	59.6(3)
C2-C7-C6	119.7(5)	O3^{3}-C18-O3	119.3(7)
C9-C8-C5	121.0(5)	O3-C18-C17	120.4(3)
C10-C8-C5	118.3(5)		

$^{1)}$ – 1 – *x*, – *y*, –1– *z*; $^{2)}$ – ½ + *x*, – ½ –*y*, ½ + *z*; $^{3)}$ – 3/2 – *x*, –1–*y*, +*z*

Table S3. Bond distances (Å) and angles(°) for shI_3972_BeDa.

Nd1-O1	2.558(6)	C10-C24	1.469(13)
Nd1-O1*w*	2.477(6)	C11-C12	1.405(12)
Nd1-O2	2.535(6)	C11-C16	1.514(11)
Nd1-O2*w*	2.515(6)	C12-C13	1.409(12)
Nd1-O3^{1}	2.464(6)	C12-C17	1.505(12)
Nd1-O3^{2}	2.947(6)	C13-C14	1.488(13)
Nd1-O42	2.454(6)	C17-C18	1.406(13)
Nd1-O5^{3}	2.415(6)	C17-C22	1.350(13)
Nd1-O6^{4}	2.395(6)	C18-C19	1.389(13)
Nd1-C1	2.928(8)	C19-C20	1.334(13)
O1-C1	1.291(11)	C20-C21	1.396(14)
O2-C1	1.252(10)	C20-C23	1.478(12)
O3-C23	1.246(11)	C21-C22	1.387(14)

O4-C23	1.268(10)	C24-C25	1.421(13)
O5-C30	1.247(10)	C24-C29	1.378(14)
O6-C30	1.260(11)	C25-C26	1.391(14)
C1-C2	1.472(11)	C26-C27	1.354(13)
C2-C3	1.392(13)	C27-C28	1.401(12)
C2-C7	1.387(13)	C27-C30	1.503(13)
C3-C4	1.399(13)	C28-C29	1.396(13)
C4-C5	1.402(14)	O7-C31	1.239(15)
C5-C6	1.358(13)	N1-C31	1.331(17)
C5-C8	1.509(11)	N1-C32	1.442(17)
C6-C7	1.379(12)	N1-C33	1.457(17)
C8-C9	1.442(13)	O8-C34	1.25(5)
C8-C13	1.392(12)	N2-C34	1.41(5)
C9-C10	1.388(12)	N2-C35	1.40(5)
C9-C15	1.524(13)	N2-C36	1.37(5)
C10-C11	1.421(12)		

[1)] ½ – *x*, ½ + *y*, 3/2 – *z*; [2)] – ½ + *x*, ½ – *y*, ½ + *z*; [3)] ½ + *x*, 3/2 – *y*, ½ + *z*; [4)] ½ – *x*, – ½ + *y*, 3/2 – *z*

C1-O1-Nd1	93.2(5)	C13-C12-C17	118.4(8)
C1-O2-Nd1	95.2(5)	C8-C13-C12	118.6(8)
$Nd1^1$-O3-$Nd1^2$	114.0(2)	C8-C13-C14	119.8(8)
C23-O3-$Nd1^1$	160.9(6)	C12-C13-C14	121.5(8)
C23-O3-$Nd1^2$	84.6(5)	C18-C17-C12	119.4(9)
C23-O4-$Nd1^2$	108.0(5)	C22-C17-C12	123.9(9)
C30-O5-$Nd1^3$	126.4(6)	C22-C17-C18	116.7(9)
C30-O6-$Nd1^4$	173.4(6)	C19-C18-C17	121.8(10)
O1-C1-Nd1	60.7(4)	C20-C19-C18	120.1(9)
O1-C1-C2	119.3(8)	C19-C20-C21	119.4(9)

O2-C1-Nd1	59.6(4)	C19-C20-C23	121.0(9)
O2-C1-O1	120.3(8)	C21-C20-C23	119.2(8)
O2-C1-C2	120.4(8)	C22-C21-C20	120.0(9)
C2-C1-Nd1	178.6(6)	C17-C22-C21	122.0(10)
C3-C2-C1	121.6(9)	O3-C23-O4	120.5(8)
C3-C2-C7	117.4(9)	O3-C23-C20	121.4(8)
C7-C2-C1	121.0(9)	O4-C23-C20	118.0(8)
C2-C3-C4	120.9(9)	C25-C24-C10	123.3(9)
C3-C4-C5	120.1(9)	C29-C24-C10	119.6(9)
C4-C5-C8	119.2(9)	C29-C24-C25	117.0(9)
C6-C5-C4	118.4(9)	C26-C25-C24	119.5(10)
C6-C5-C8	122.5(9)	C27-C26-C25	122.4(9)
C5-C6-C7	121.7(9)	C26-C27-C28	119.4(9)
C6-C7-C2	121.4(9)	C26-C27-C30	121.5(8)
C9-C8-C5	119.2(8)	C28-C27-C30	119.0(8)
C13-C8-C5	119.7(8)	C29-C28-C27	118.5(9)
C13-C8-C9	121.0(8)	C24-C29-C28	123.1(10)
C8-C9-C15	121.1(9)	O5-C30-O6	124.0(8)
C10-C9-C8	118.8(8)	O5-C30-C27	117.5(8)
C10-C9-C15	119.8(9)	O6-C30-C27	118.5(8)
C9-C10-C11	121.2(8)	C31-N1-C32	123.1(14)
C9-C10-C24	118.3(8)	C31-N1-C33	116.3(12)
C11-C10-C24	120.4(8)	C32-N1-C33	120.5(14)
C10-C11-C16	120.3(8)	O7-C31-N1	123.4(15)
C12-C11-C10	118.4(8)	C34-N2-C35	126(4)
C12-C11-C16	121.2(8)	C36-N2-C34	107(4)
C11-C12-C13	122.0(8)	C36-N2-C35	126(4)
C11-C12-C17	119.6(8)	O8-C34-N2	113(4)

[1] ½ – *x*, – ½ + *y*, 3/2 – *z*; [2] ½ + *x*, ½ – *y*, – ½ + *z*; [3] – ½ + *x*, 3/2 – *y*, – ½ + *z*; [4] ½ – *x*, ½ + *y*, 3/2 – *z*;

Table S4. Bond distances (Å) and angles(º) for shI_3984_BeDa

La1-O1	2.503(7)	C5-C8	1.487(11)
La1-O1^{1}	3.017(7)	C6-C7	1.371(12)
La1-O1*w*	2.545(7)	C8-C9	1.396(10)
La1-O2^{1}	2.497(7)	C8-C10	1.394(13)
La1-O3^{2}	2.611(6)	C9-C12	1.52(2)
O1-C1	1.291(11)	C10-C11	1.414(11)
O2-C1	1.237(11)	C10-C13	1.513(12)
O3-C18	1.276(8)	C11-C14	1.506(19)
C1-C2	1.483(11)	C14-C15	1.379(12)
C2-C3	1.349(11)	C14-C15^{3}	1.380(12)
C2-C7	1.427(12)	C15-C16	1.377(14)
C3-C4	1.385(12)	C16-C17	1.381(11)
C4-C5	1.381(13)	C17-C18	1.506(17)
C5-C6	1.388(13)		

$^{1)}$ ½ + *x*, + *y*, 1 – *z*; $^{2)}$ + *x*, ½ – *y*, ½ – *z*; $^{3)}$ 3/2 – *x*, – *y*, + *z*

La1-O1-La1^{1}	116.0(2)	C10-C8-C5	117.6(7)
C1-O1-La1^{1}	83.4(5)	C10-C8-C9	121.7(8)
C1-O1-La1	159.9(6)	C8-C9-C8^{3}	118.7(11)
C1-O2-La1^{1}	110.0(6)	C8^{3}-C9-C12	120.6(6)
C18-O3-La1^{2}	93.6(5)	C8-C10-C11	118.8(8)
O1-C1-C2	120.0(8)	C8-C10-C13	121.1(8)
O2-C1-O1	120.6(8)	C11-C10-C13	120.1(9)
O2-C1-C2	119.4(8)	C10-C11-C10^{3}	120.4(12)
C3-C2-C1	123.8(8)	C10-C11-C14	119.8(6)
C3-C2-C7	117.6(7)	C10^{3}-C11-C14	119.8(6)
C7-C2-C1	118.3(7)	C15-C14-C11	120.5(6)

C2-C3-C4	122.1(8)	C15[3]-C14-C15	119.1(12)
C5-C4-C3	121.0(9)	C14-C15-C16	120.5(10)
C4-C5-C6	117.5(8)	C17-C16-C15	120.6(10)
C4-C5-C8	120.3(9)	C16[3]-C17-C16	118.8(12)
C6-C5-C8	122.0(8)	C16-C17-C18	120.6(6)
C7-C6-C5	121.8(9)	O3-C18-La1[2]	61.0(5)
C6-C7-C2	119.9(8)	O3[3]-C18-O3	122.1(11)
C9-C8-C5	120.7(8)	O3-C18-C17	119.0(5)

[1)] $1 - x, 1 - y, 1 - z$; [2)] $3/2 - x, -½ + y, ½ - z$; [3)] $3/2 - x, -y, +z$
